# Virtual Screening and Molecular Dynamics Simulations Identifying Natural Product-Derived Cathepsin K Inhibitors as Potential Therapeutics for Osteoporosis

**DOI:** 10.3390/ijms27188258

**Published:** 2026-09-16

**Authors:** Thitinun Tarathipayakul, Yuvaraj Ravikumar, Pattaranee Srichairatanakool, Jittasak Khowsathit, Somdet Srichairatanakool

**Affiliations:** 1 Department of Orthopedic, School of Medicine, University of Phayao, Phayao 56000, Thailand; thitinun.tar@up.ac.th; 2 Department of Basic Sciences, School of Sciences and Humanities, SR University, Warangal 506371, India; yuvaraj.r@sru.edu.in; 3 Department of Biochemistry, Faculty of Medicine, Chiang Mai University, Chiang Mai 50200, Thailand; jittasak.khowsathit@cmu.ac.th; 4 Department of Anesthesiology, School of Medicine, University of Phayao, Phayao 56000, Thailand; pattaranee.sr@up.ac.th

**Keywords:** osteoporosis, cathepsin K, virtual screening, molecular docking, molecular simulations, dynamics

## Abstract

Osteoporosis is a prevalent skeletal disorder characterized by excessive bone resorption and an increased risk of fragility fracture. Cathepsin K (CatK), a lysosomal cysteine protease predominantly expressed in osteoclasts, is an established target for anti-resorptive drug development. Here, an integrated computational workflow comprising virtual screening, molecular docking, 300 ns molecular dynamics (MD) simulations, molecular mechanics/Poisson–Boltzmann surface area (MM/PBSA) calculations, and drug-likeness/ADMET prediction was used to prioritize natural product-derived CatK ligands from the MEGxM database. Among approximately 6500 screened compounds, TOP1 (PubChem ID: 97043052) and TOP2 (PubChem ID: 135765825) showed docking scores of −8.2 and −7.8 kcal/mol, respectively, compared with −6.6 kcal/mol for the comparative reference ligand (STD). Both compounds satisfied Lipinski’s rule of five; however, their predicted ADMET profiles were mixed, including negative predictions for human intestinal absorption for both compounds and compound-specific metabolic and toxicity liabilities. The 300 ns MD trajectories indicated broadly stable CatK ligand complexes. TOP1 showed slightly lower mean RMSD)/RMSF values than TOP2. In contrast, TOP2 exhibited more favorable MM/PBSA binding free energies than TOP1 at both analyzed intervals (−83.45 ± 10.06 and −80.74 ± 10.18 kJ/mol for TOP2 versus −74.28 ± 10.08 and −57.21 ± 15.04 kJ/mol for TOP1), while STD showed the most favorable MM/PBSA binding free energies overall. Collectively, TOP1 and TOP2 exhibited complementary computational profiles and should therefore be regarded as candidates for biochemical and cellular validation rather than confirmed CatK inhibitors or orally suitable drug leads.

## 1. Introduction

Osteoporosis is a chronic skeletal disorder characterized by reduced bone mineral density (BMD), deterioration of bone microarchitecture, and increased susceptibility to fragility fractures. It represents a major global public health challenge, particularly among postmenopausal women and older adults, resulting in substantial increases in morbidity rates, mortality rates, and healthcare costs worldwide [1,2]. The pathogenesis of osteoporosis is primarily associated with an imbalance between bone formation by osteoblasts and bone resorption by osteoclasts, leading to progressive bone loss and structural deterioration [3]. Bone remodeling is a highly regulated physiological process controlled by complex signaling pathways involving the receptor activator of nuclear factor kappa B (RANK), the receptor activator of nuclear factor kappa B ligand (RANKL), osteoprotegerin (OPG), estrogen, and inflammatory cytokines [4]. Following menopause, estrogen deficiency promotes RANK ligand (RANKL) expression while reducing OPG production, thereby enhancing osteoclast differentiation and activity while accelerating bone resorption [5]. Consequently, excessive osteoclastic activity has become a major therapeutic target in osteoporosis management. Current pharmacological therapies for osteoporosis include bisphosphonates, selective estrogen receptor modulators, denosumab, parathyroid hormone analogs, and romosozumab [6]. Although these agents effectively reduce fracture risk, several limitations remain, including gastrointestinal intolerance, osteonecrosis of the jaw, atypical femoral fractures, cardiovascular concerns, high treatment costs, and reduced patient adherence during long-term therapy [6,7]. Therefore, the identification of novel therapeutic targets and safer anti-resorptive agents remains an important research priority.

Cathepsin K (CatK; EC 3.4.22.38) is a lysosomal cysteine protease predominantly expressed in osteoclasts and is recognized as the principal enzyme responsible for degradation of type I collagen, the major organic component of bone matrix [8,9]. During bone resorption, CatK is secreted into the resorption lacunae, where it efficiently degrades collagen fibrils and other extracellular matrix proteins under acidic conditions [10]. Genetic deficiency of CatK causes pycnodysostosis, a rare skeletal disorder characterized by impaired bone resorption and increased bone density, highlighting the critical physiological role of this enzyme in bone remodeling [11]. Furthermore, numerous experimental and clinical studies have demonstrated that CatK inhibition suppresses osteoclastic bone resorption while preserving osteoblast-mediated bone formation, making CatK an attractive therapeutic target for osteoporosis treatment [12].

Several synthetic CatK inhibitors, including odanacatib, balicatib, relacatib, and ONO-5334, have been developed and evaluated in preclinical and clinical studies [12,13]. Among these, odanacatib demonstrated significant improvements in BMD values and fracture reduction in Phase III clinical trials. However, its development was discontinued because of concerns regarding increased cardiovascular and cerebrovascular adverse events [14]. Similarly, other CatK inhibitors encountered challenges related to off-target effects, insufficient selectivity, or unfavorable safety profiles, all of which prevented regulatory approval [12,14]. Despite decades of research, no CatK inhibitor has yet reached the pharmaceutical market. Natural products have historically served as an important source of therapeutic agents owing to their structural diversity, biological activities, and generally favorable safety profiles [15]. Numerous phytochemicals, including flavonoids, polyphenols, xanthones, and terpenoids, have demonstrated anti-osteoporotic properties through antioxidant, anti-inflammatory, and osteoclast-modulating mechanisms [16]. Recent studies have further suggested that certain natural compounds may directly inhibit CatK activity, highlighting their potential as alternative or complementary therapeutic agents for osteoporosis [12,17].

Advances in computational drug discovery have significantly accelerated the identification of important bioactive compounds. Structure-based virtual screening, molecular docking, molecular dynamics simulations, and in silico pharmacokinetic analyses enable the rapid evaluation of large chemical libraries while reducing the cost and time associated with experimental screening [18]. These computational approaches have become indispensable tools for identifying potential enzyme inhibitors and prioritizing compounds for subsequent biological validation.

The Medicinal and Economic Plants Genome Database (MEGxM) contains thousands of natural product-derived compounds originating from medicinal plants and represents a valuable resource for discovering novel bioactive molecules. However, the CatK inhibitory potential of many compounds within this database remains unexplored. Therefore, the present study employed an integrated computational workflow consisting of virtual screening, molecular docking, molecular dynamics simulations, and free energy calculations, as well as absorption, distribution, metabolism, excretion, and toxicity (ADMET) predictions, to identify potential CatK inhibitors from the MEGxM database. The findings may provide novel lead compounds for further experimental evaluation and the future development of safer anti-osteoporotic therapeutics.

## 2. Results

### 2.1. Molecular Docking

**Figure 1:**
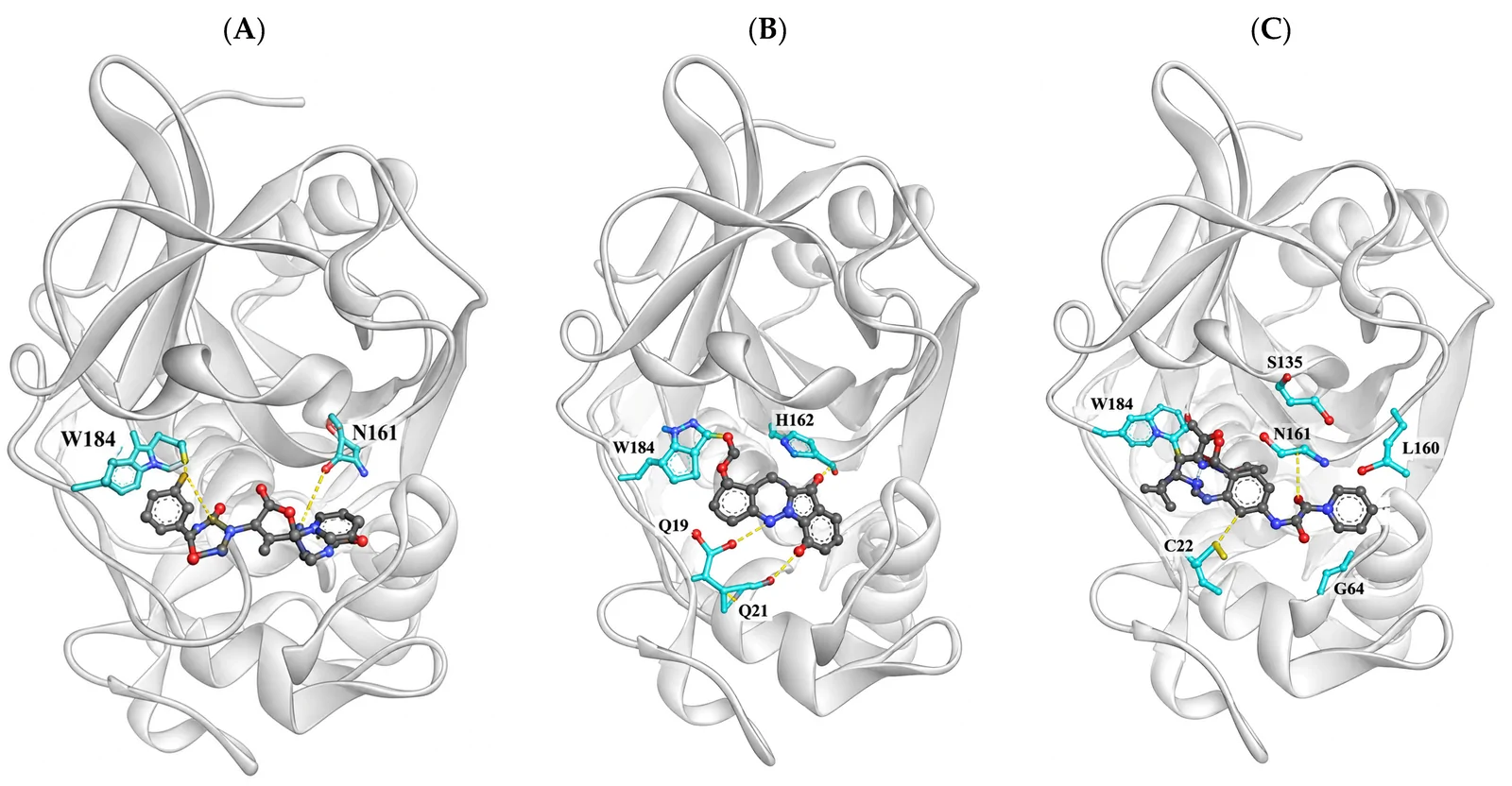
Three-dimensional (3D) interaction diagrams of selected top-ranked compounds docked in the binding pocket of cathepsin K (PubChem ID: 2ATO). (**A**) 2ATO-TOP1 (PubChem ID: 97043052); (**B**) 2ATO-TOP2 (PubChem: 135765825; (**C**) 2ATO-STD (PubChem ID: 44194893). Protein structures have been depicted in illustration models. Ligands are represented as stick models, while interacting amino acid residues are presented as sticks. Hydrogen bonds have been depicted as yellow lines in 2ATO-TOP1 and green lines in 2ATO-TOP2 and 2ATO-STD, while hydrophobic interactions (alkyl and π–alkyl interactions) are indicated by violet and grey dashed lines. Residue labels correspond to the amino acids present within the active site cavity of 2ATO.

The MEGxp database, containing 6539 natural product-derived compounds, was selected for virtual screening against CatK (PDB ID: 2ATO). Among them, 1450 molecules that passed Lipinski’s rule of five were chosen for molecular docking. The reference compound (STD; PubChem ID: 44194893) was included for comparison. The co-crystallized ligand and the CatK-binding pocket were inspected before docking, and residual numbering was standardized throughout the manuscript according to the 2ATO sequence. The docked compounds were ranked by docking score. TOP1 exhibited a docking score of −8.2 kcal/mol and TOP2 −7.8 kcal/mol, compared with −6.6 kcal/mol for STD. The docking results and contacts for TOP1-TOP5 are summarized in Table 1 and representative three-dimensional (3D) interactions of TOP1, TOP2, and STD with CatK are shown in Figure 1A, Figure 1B and Figure 1C, respectively.

**Figure 2:**
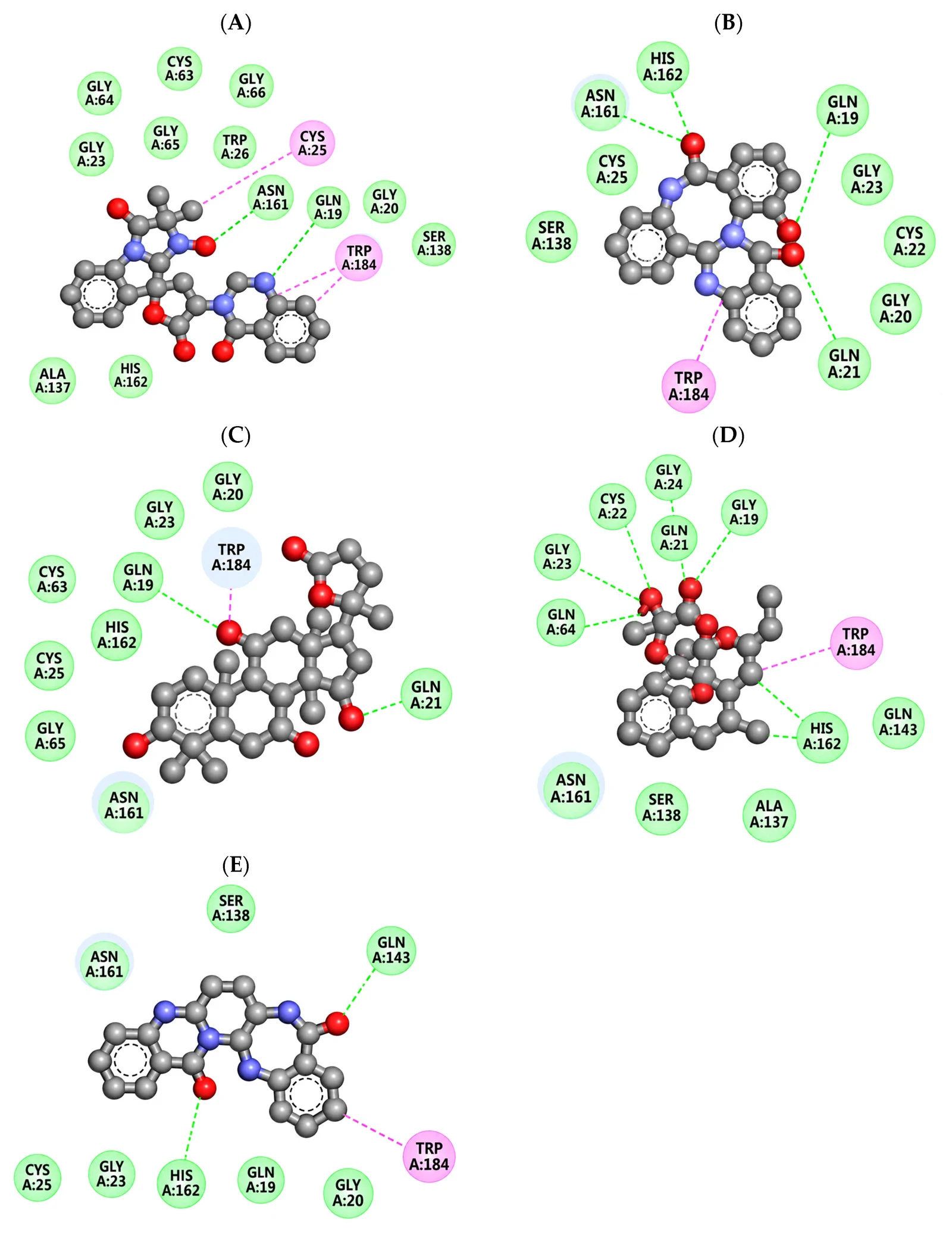
Two-dimensional (2D) molecular interaction maps of the top-ranked similarity-selected compounds (TOP1–TOP5) bound to the active site of the CatK. Hydrophobic interactions and hydrogen bond contacts between each ligand and the surrounding amino acid residues are illustrated. (**A**) TOP1, (**B**) TOP2, (**C**) TOP3, (**D**) TOP4, (**E**) TOP5.

The docking interactions were re-checked against Table 1, and residue numbering was standardized to the CatK 2ATO sequence. TOP1 formed hydrogen bond contacts with Q19 and N161 and additional non-covalent contacts with W184 and C25. TOP2 formed hydrogen bond contacts with C25, H162, Q21, and Q19, with an additional contact involving W184. Previously stated residues F362, H435, and R370 were inconsistent with the 2ATO sequence and were removed. Interactions of TOP1-TOP5 are summarized in Table 1 and illustrated in Figure 2.

### 2.2. Prediction of Pharmacokinetics and Toxicological Profiles

The pharmacokinetics, including the ADME of the TOP1 and TOP2 compounds, were assessed by the SwissADME web-based server. The crucial parameters that determined drug-likeness, such as molecular weight (MW), hydrogen bond acceptors and donors, and topological polar surface area (TPSA), were evaluated. As shown in Table 2, both compounds showed MW of 432.14 g/mol (TOP1) and 355.10 g/mol (TOP2). The numbers of rotatable bonds, which indicate molecular flexibility, were 1 and 0 for the TOP1 and TOP2 compounds, respectively. In TOP1, the numbers (*n*) of the H-bond acceptors (HA) and the H-bond donors (HD) were 9 and 1, respectively, while in TOP2, the corresponding values were 6 and 2. The TPSA, which generally reflects bioavailability and membrane permeability, showed values of 104.97 and 84.22 A2. Overall, both TOP1 and TOP2 satisfied Lipinski’s rule of five without violations; however, this physicochemical criterion alone does not establish favorable oral absorption or bioavailability.

The ADMET results were interpreted strictly according to the prediction outputs summarized in Table 3. The predicted Caco-2 permeability values were −5.489 and −4.892 for TOP1 and TOP2, respectively, while MDCK permeability was reported as 0.0 for both. Both were predicted to be negative for BBB permeability and P-glycoprotein (P-gp1) substrate status, whereas TOP2, but not TOP1, was predicted to inhibit a P-gpI. The CYP predictions also differed between the compounds. TOP1 was predicted to be negative for CYP2D6 substrate status and for inhibition of CYP1A2, CYP2C19, CYP2C9, CYP2D6 and CYP3A4, but positive for CYP3A4 substrate status. In contrast, TOP2 was predicted to be a substrate for CYP2D6 and CYP3A4 and showed positive predictions for CYP1A2 and CYP3A4 inhibition. The numerical outputs for Ames mutagenicity, genotoxicity, hepatotoxicity, carcinogenicity, and HEK293 cytotoxicity are presented in Table 3 as model-derived prediction scores and should not be interpreted as experimentally confirmed toxic or non-toxic classifications. Overall, the predicted ADMET profiles of TOP1 and TOP2 were mixed and indicated potential limitations related to intestinal absorption, metabolic interaction, and predicted toxicity endpoints; therefore, these computational results do not support definitive claims of favorable pharmacokinetic and safety profiles without experimental validation.

### 2.3. Molecular Dynamics Simulation

The structural and dynamic behavior of apo CatK and the TOP1–CatK, TOP2–CatK, and STD–CatK complexes during the 300-ns MD simulations was evaluated using multiple complementary trajectory analyses. The results of the root mean square deviation (RMSD), root mean square fluctuation (RMSF), radius of gyration (Rg), hydrogen-bond, solvent-accessible surface area (SASA), principal component analysis (PCA), free-energy landscape (FEL), secondary-structure, dynamic cross-correlation matrix (DCCM), and molecular mechanics Poisson-Boltzmann surface area (MM/PBSA) analyses are presented below.

#### 2.3.1. RMSD Analysis

**Figure 3:**
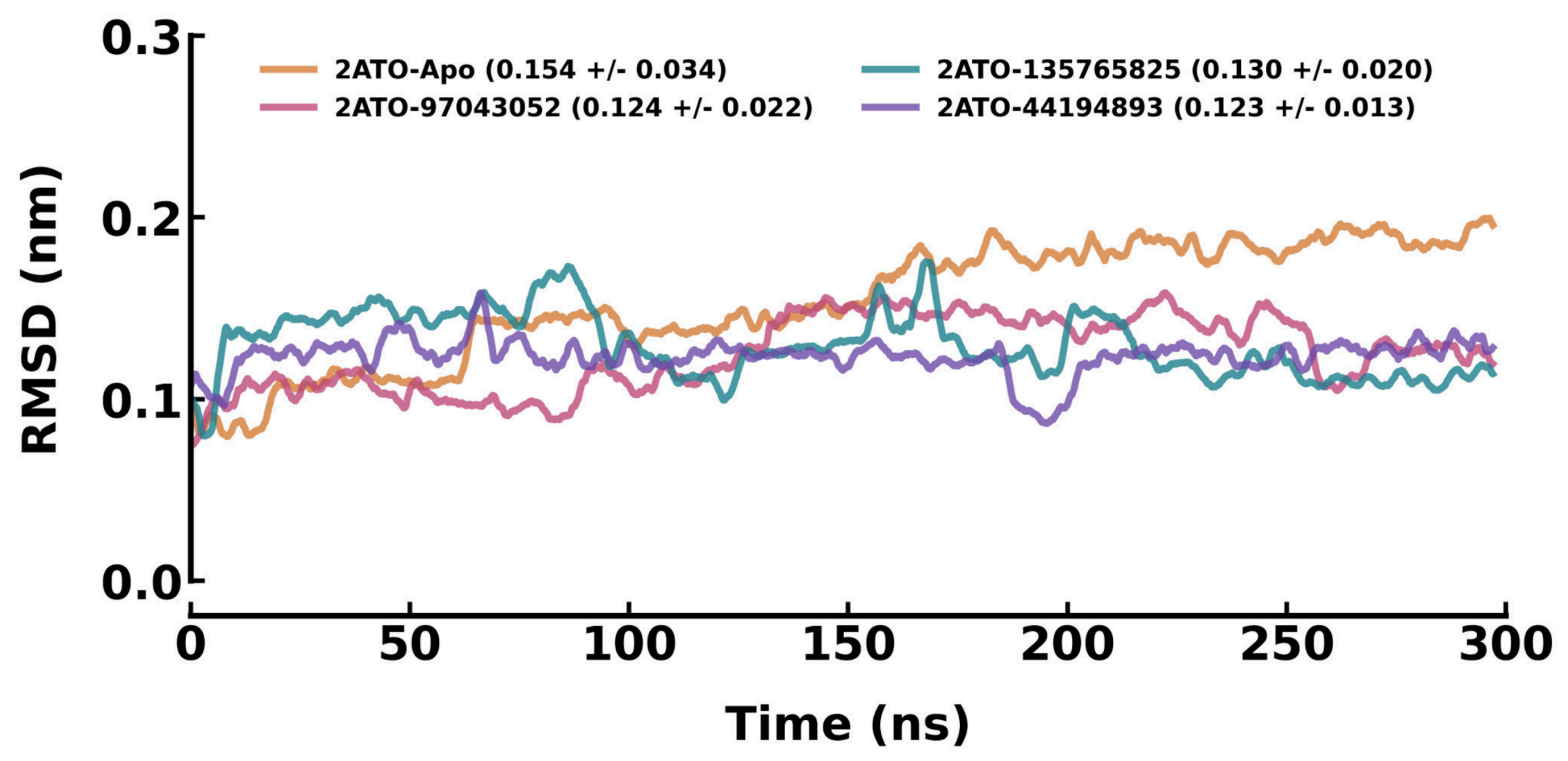
Root mean square deviation (RMSD) profiles of the apo CatK domain and protein–ligand complexes during the 300 ns molecular dynamics simulation. The RMSD values were calculated relative to the initial structures to evaluate the structural stability of the apo protein and ligand-bound complexes over the simulation period. Data are presented as mean RMSD ± standard deviation (SD) values.

The RMSD system for the CatK Cα atoms was analyzed to compare the conformational behavior of the apo CatK and ligand-bound systems during the 300 ns molecular dynamics simulations (Figure 3). The mean RMSD values calculated over the molecular trajectory were 0.15 ± 0.03 nm for apo CatK, 0.12 ± 0.02 nm for the TOP1-CatK complex, 0.13 ± 0.02 nm for TOP2-CatK complex, and 0.12 ± 0.01 nm for STD-CatK complex, as summarized in Supplementary Table S1. These values indicate broadly stable trajectories for the ligand-bound systems, with TOP1-CatK and STD-CatK showing similar mean RMSD values and TOP2-CatK showing a slightly higher average RMSD. Accordingly, the RMSD data do not support the conclusion that TOP1-CatK was more structurally stable than STD-CatK. The Kernel density distributions (KDEs) and temporal RMSD heatmap in Figure S2 provide complementary visualization of these trajectory distributions, showing narrower RMSD distributions and lower structural variability for the ligand-bound systems than for apo CatK.

#### 2.3.2. RMSF Analysis

**Figure 4:**
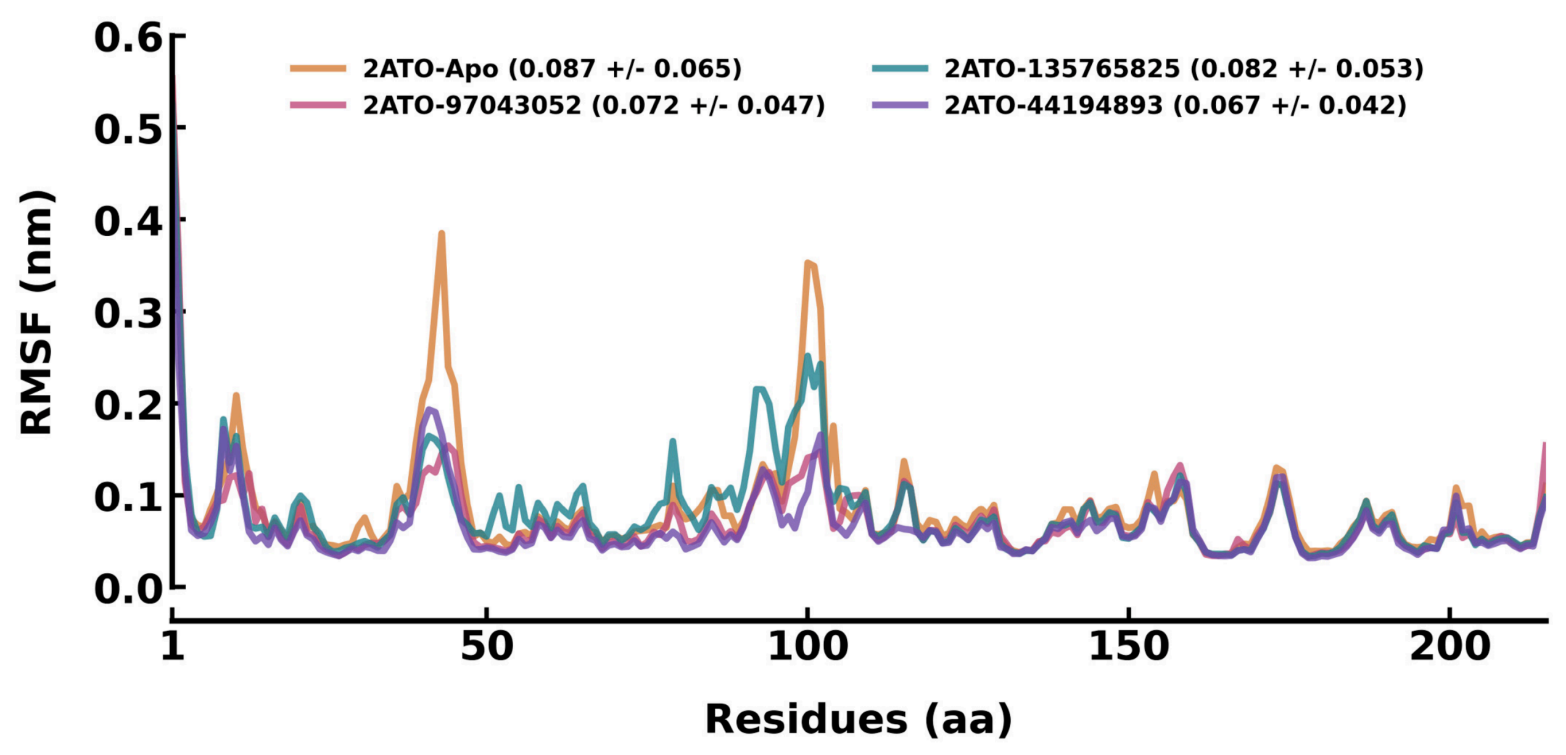
Root mean square fluctuation (RMSF) analysis of the apo CatK domain and protein–ligand complexes during the 300 ns molecular dynamics simulation. RMSF values were calculated for each amino acid residue to evaluate local structural flexibility and conformational fluctuations in the apo protein and ligand-bound states. The average RMSF ± SD values for each system are indicated in the legend.

Accordingly, to identify the definitive regions that were susceptible to distortion in the CatK, RMSF was analyzed for the aforementioned systems. Comparable and similar patterns were noted in all the ligand-bound complexes. The fewest number of fluctuations with the lowest average values were observed in the TOP1-CatK (0.07 ± 0.05 nm), while slightly higher fluctuations were measured in TOP2 (0.08 ± 0.05 nm) when compared with the reference compound (STD − 0.07 ± 0.04 nm). Ligand-free CatK showed a higher average RMSF value of 0.09 ± 0.06 nm when compared with the protein backbone (Figure 4). These findings reveal that TOP1 and TOP2 yielded more stable proteins with minimal residue-level fluctuations. Furthermore, it has been suggested that both TOP1 and TOP2 compounds remained stably bound within the CatK active site, demonstrating the least amount of divergence from their initial conformations, as has been verified by trajectory assessment. These observations were further supported by the RMSF probability density distributions and residue-wise RMSF heatmap, which confirmed the reduced structural flexibility of the ligand-bound complexes relative to the apo protein (Figure S3).

#### 2.3.3. Rg Analysis

**Figure 5:**
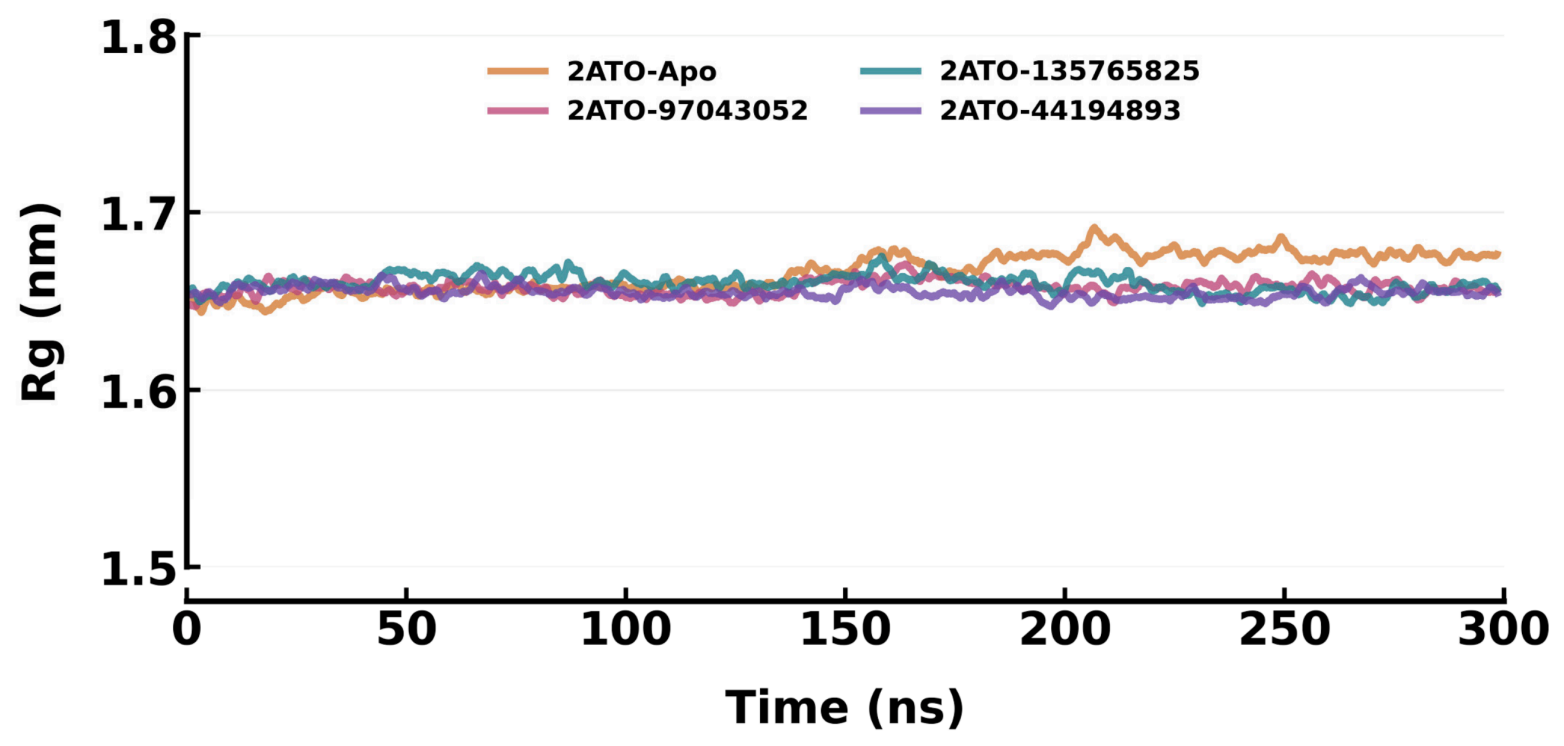
Radius of gyration (Rg) profiles of the apo CatK domain and protein–ligand complexes during the 300 ns molecular dynamics simulation. The Rg values were calculated throughout the simulation to evaluate the structural compactness and overall folding stability of the apo protein and ligand-bound complexes. Stable Rg values indicate maintenance of protein compactness during the simulation period.

Rg provides insight into protein folding behavior and variations in compactness by quantifying the displacement of atoms from their initial coordinates over the course of the simulations. A lower Rg value generally indicates tightly packed but intact packing of the atoms, which is ultimately indicative of the presence of well-organized secondary structures. Accordingly, the Rg values for all the systems were investigated and are depicted in Figure 5. The Rg values of 1.65–1.68 nm in the Apo form indicated a time evolution-based variation in compactness. The predicted average Rg values were 1.67 ± 0.01 nm for Apo and 1.66 ± 0.01 nm for all the remaining ligand-bound complexes (i.e., TOP1-CatK, TOP2-CatK, and STD-CatK). These results imply that compactness was mostly steady with all the ligand-bound complexes, with the mean values being quite similar and close to that of the STD compound. Nonetheless, a meager fluctuation of the Rg values was observed after 150 ns in the Apo system. Taken together, the Rg analysis indicated a well-maintained, compact folded protein after binding to the TOP1 and TOP2 compounds. These findings were further supported by the Rg probability density distributions, which showed narrower Rg distributions and highly similar mean Rg values for the ligand-bound complexes relative to the apo protein, indicating stable structural compactness throughout the simulation (Figure S4).

#### 2.3.4. H-Bond Analysis

**Figure 6:**
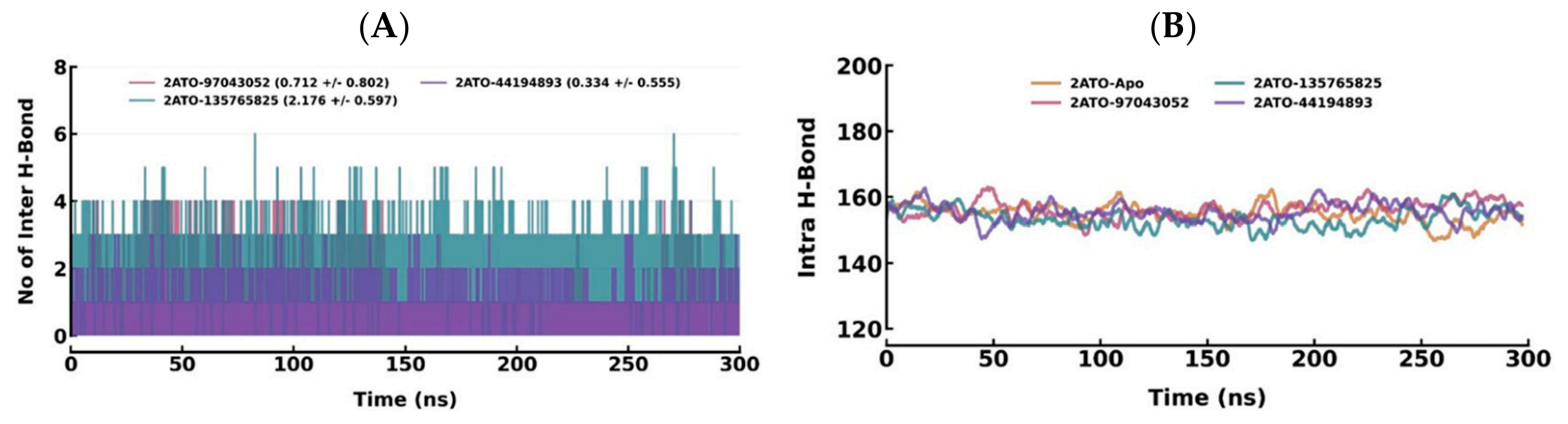
Hydrogen bond analysis of the apo CatK domain and protein–ligand complexes during the 300 ns molecular dynamics simulation. (**A**) Number of intermolecular hydrogen bonds formed between the ligands and the apo CatK domain throughout the simulation. Average hydrogen bond numbers for the TOP1, TOP2, and STD complexes are indicated in the legend. (**B**) Number of intramolecular hydrogen bonds within the apo protein and ligand-bound complexes over the simulation period. Stable hydrogen bond profiles indicate preservation of protein structural integrity and favorable protein–ligand interactions.

Hydrogen bond analysis was used to characterize intermolecular ligand CatK contacts and intramolecular protein hydrogen bonds during the 300 ns simulations (Figure 6). Intermolecular hydrogen bond counts varied over time; consequently, maximum instantaneous counts were not interpreted as direct measures of binding affinity. Temporal intermolecular hydrogen bond occupancy and probability distributions are shown in Figure S5, and residue-wise hydrogen bond contacts are presented in Figure S6. The mean intramolecular hydrogen bond counts were 155.02 ± 5.96 for apo CatK, 156.23 ± 5.78 for TOP1-CatK, 153.31 ± 6.00 for TOP2-CatK, and 155.38 ± 5.88 for STD-CatK. These values are counts and therefore have no length unit. Overall, the profiles indicate preservation of the protein hydrogen bond network in all systems but do not by themselves establish superior ligand binding. Representative structural snapshots of TOP1-CatK and TOP2-CatK are provided in Figures S8 and S9, respectively.

#### 2.3.5. SASA Analysis

**Figure 7:**
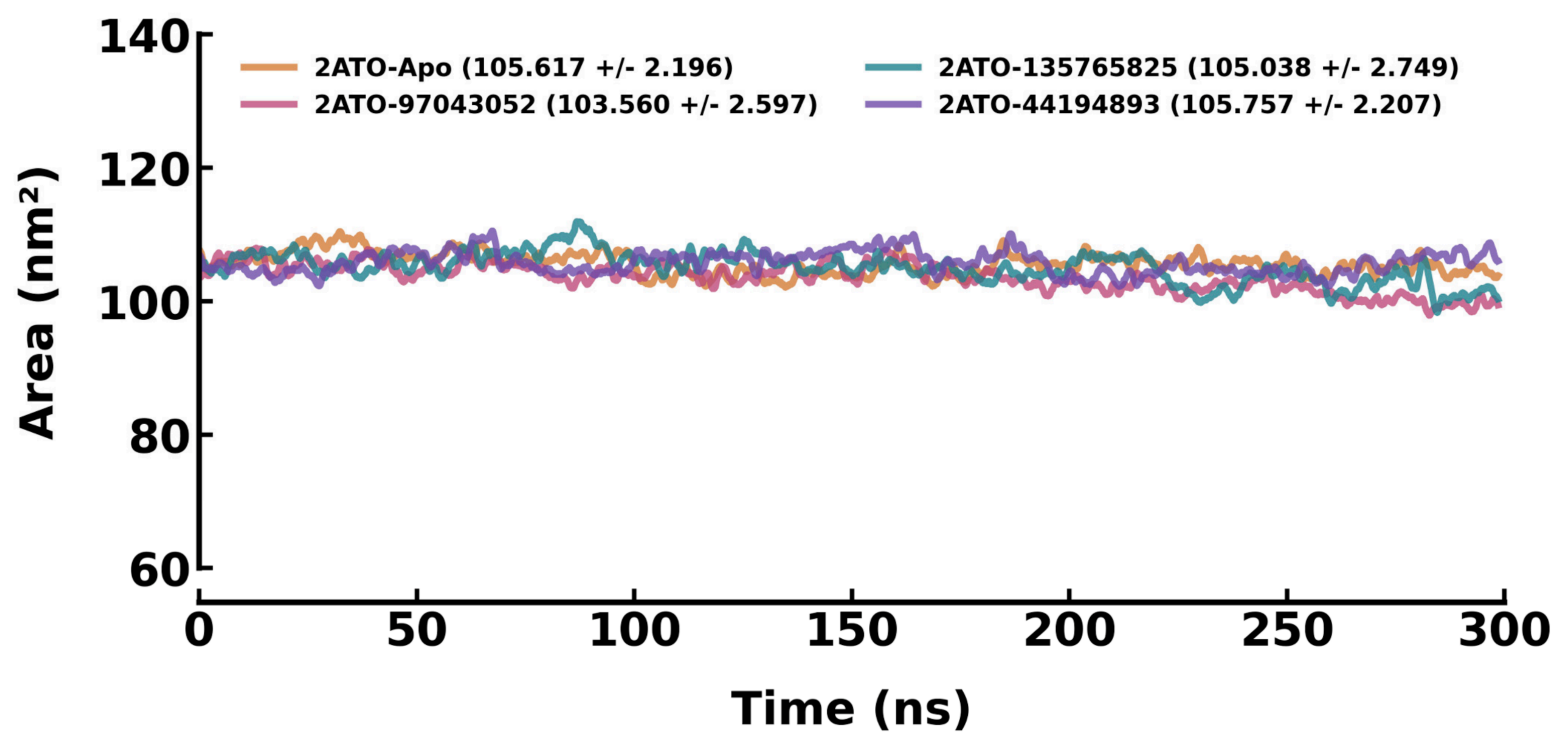
Solvent-accessible surface area (SASA) profiles of the apo CatK domain and protein–ligand complexes during the 300 ns molecular dynamics simulation. SASA values were calculated throughout the simulation to evaluate changes in protein surface exposure and conformational stability upon ligand binding. Stable SASA values indicate maintenance of the overall protein structure and solvent accessibility during the simulation period.

SASA reflects the protein surface accessible to solvent and was evaluated throughout the simulations (Figure 7). The mean SASA values were 105.76 ± 2.21 nm^2^ for apo CatK, 103.56 ± 2.60 nm^2^ for TOP1-CatK, 105.62 ± 2.20 nm^2^ for TOP2-CatK, and 105.04 ± 2.75 nm^2^ for STD-CatK. The values were broadly comparable across systems, with TOP1 showing a modestly lower mean SASA. These data suggest no major ligand-associated change in global solvent exposure within the resolution of this analysis. The corresponding SASA probability density distributions and temporal heatmap are presented in Figure S7.

#### 2.3.6. PCA and FEL Analysis

**Figure 8:**
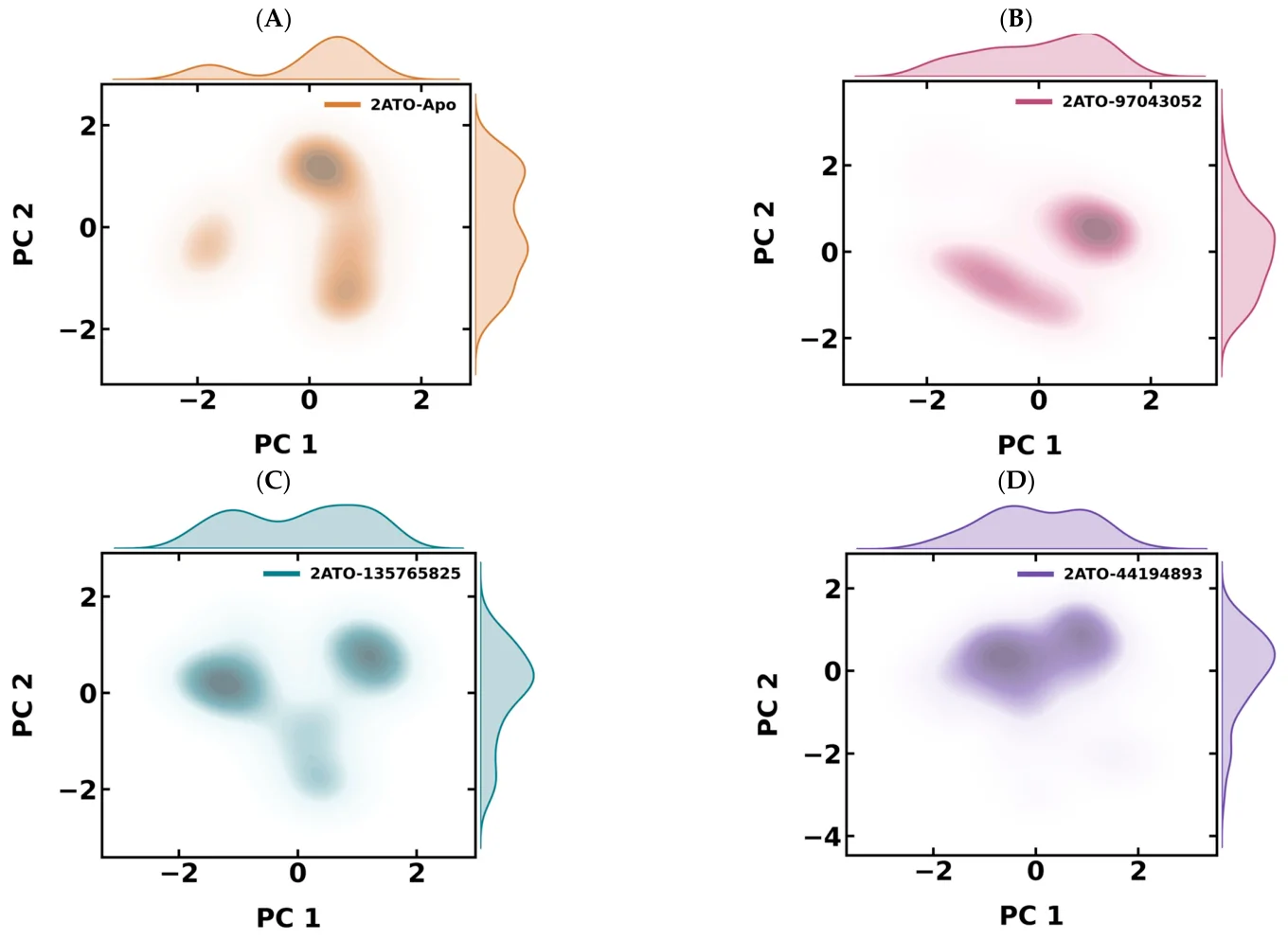
Principal component (PC) analysis of the conformational motions of apo CatK and protein–ligand complexes during the 300 ns molecular dynamics simulation. Two-dimensional projections onto PC1 and PC2 are shown for (**A**) apo CatK, (**B**) TOP1-CatK, (**C**) TOP2-CatK, and (**D**) STD-CatK (STD; PubChem ID: 44194893). The distributions represent the conformational space sampled by each system during the simulation.

The individual systems comprising Apo-CatK, TOP1-CatK, TOP2-CatK, and STD-CatK, along with their covariance matrices for atomic motions, are plotted in Figure 8. The 2D PCA projection plots for Apo reveal that multiple compact conformational groups are dispersed across the region. Maximum clusters were mainly observed in regions corresponding to positive PC1 and a wide range of PC2 values, implying the presence of many metastable states. In the TOP1-Catk system, the distinct clusters appeared elongated and were present in both the positive and negative PC2 values. This scattered distribution along both axes indicates that the TOP1 binds to CatK, resulting in a stable conformational state. Similar results were seen with the TOP2 ligand-bound systems as well. The major and distinct conformational states were observed, and, when compared with the apo system, the clusters were apparently dispersed, suggesting the existence of states attempting to attain equilibrium and conformational stability. In contrast with the TOP1 and TOP2 ligand-bound complexes, STD-CatK exhibited highly diverse and scattered clusters. In particular, a few distinct clusters were overlaid with a few dispersed points in the negative PC1 and PC2 regions. This result indicates that the evolution of the heterogeneous state was involved with the STD, as well as being indicative of a stable conformation over the 300 ns period. Taken together, the PC analysis suggests that the binding of the TOP1 and TOP2 compounds to CatK had not substantially or adversely affected the protein’s conformational landscape, as evidenced by the limited, distinct, and well-dominant clusters present across both PC1 and PC2. Furthermore, the PC1 and PC2 eigenvalues were used to explore the Gibbs energy landscape. Typically, the Gibbs free energy reflects the existence of conformational states of the protein by representing them as a color gradient from blue to red. The lower energy highly stable conformation is depicted in dark blue, while the higher energy unstable state is shown in dark red. The unstable and intermediate states are shown in yellow. The 3D plots for all the systems are depicted in Figure 8. As can be seen in the 3D plots, the TOP1 and TOP2 compounds exhibit a well-defined, sharper, deeper, and more intense lower energy state in dark blue. This suggests that ligand binding does not perturb the protein into multiple microstates but rather restricts it to energetically favorable conformational states. Similar patterns were observed in the reference STD systems, except for when there were fewer intermediate basins separated by a moderate energy landscape, suggesting the possibility of heterogeneous substates during the simulation period.

#### 2.3.7. Secondary Structure Analysis

**Figure 9:**
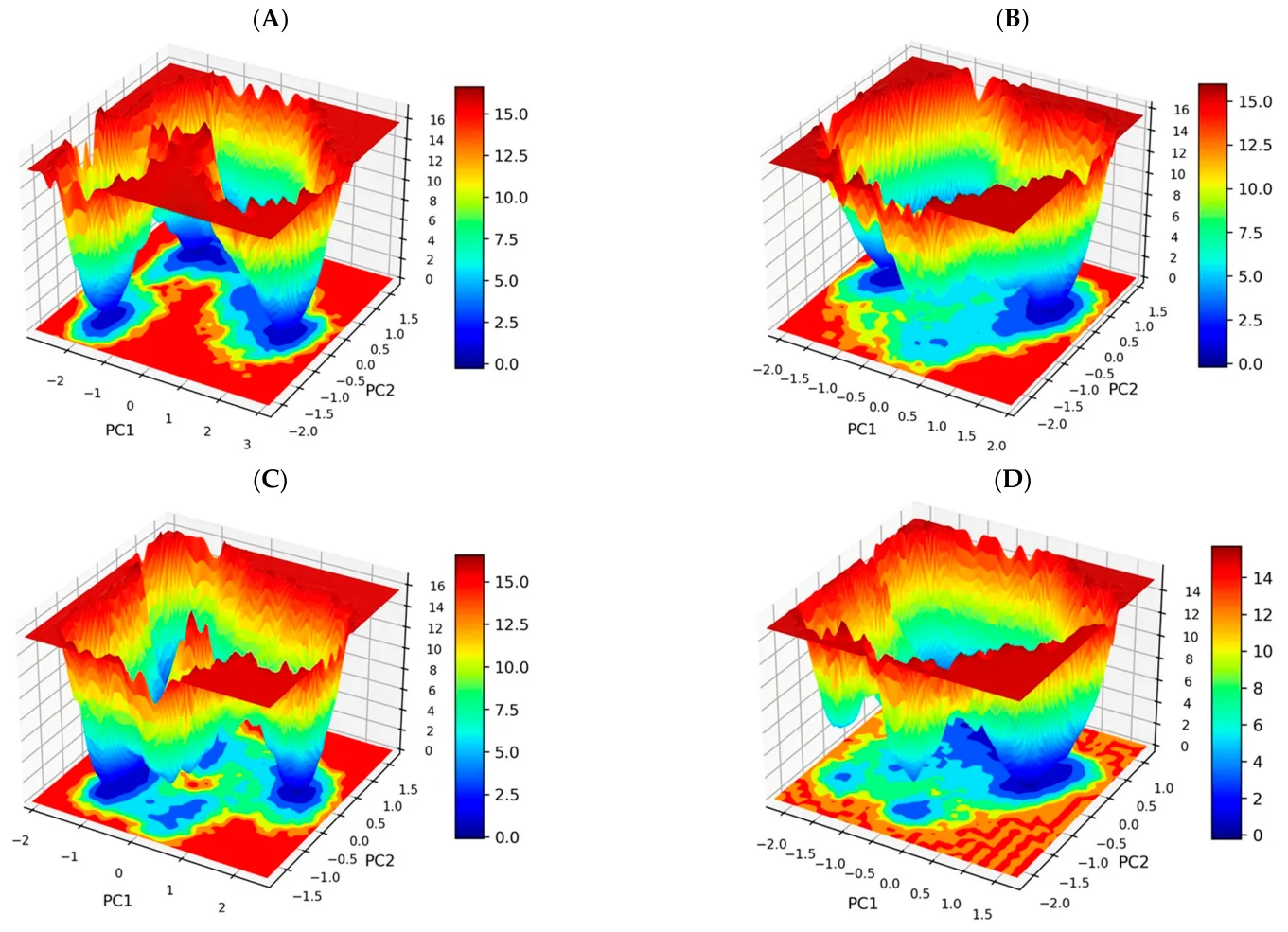
Free energy landscape (FEL) analysis of the apo CatK domain and protein–ligand complexes derived from the molecular dynamic trajectories. The free energy landscapes were constructed using the first two principal components (PC1 and PC2) obtained from the principal component analysis. (**A**) Apo CatK, (**B**) TOP1, (**C**) TOP2 complex, and (**D**) STD complex. Blue regions represent low-energy conformational states, whereas red regions correspond to higher energy states.

The secondary structure variation of the CatK protein upon binding to different ligands and in the unbound state was evaluated using the define secondary structure protein (DSSP) tool of the Groningen Machine for Chemical Simulations (GROMACS). The secondary structure evolution over the 300 ns simulation period for TOP1, TOP2, and the STD-bound CatK complexes was evaluated. The differences related to α-helices (blue color), β-sheets (red color), and turns or bends (yellow) are represented in Figure 9. With respect to the TOP1 and TOP2 bound systems, consistent red and blue colors with very few white colors were observed. This result indicates well-preserved secondary protein structures, particularly α-helices and β-sheets, suggesting that TOP1 and TOP2 binding did not affect conformational stability.

**Figure 10:**
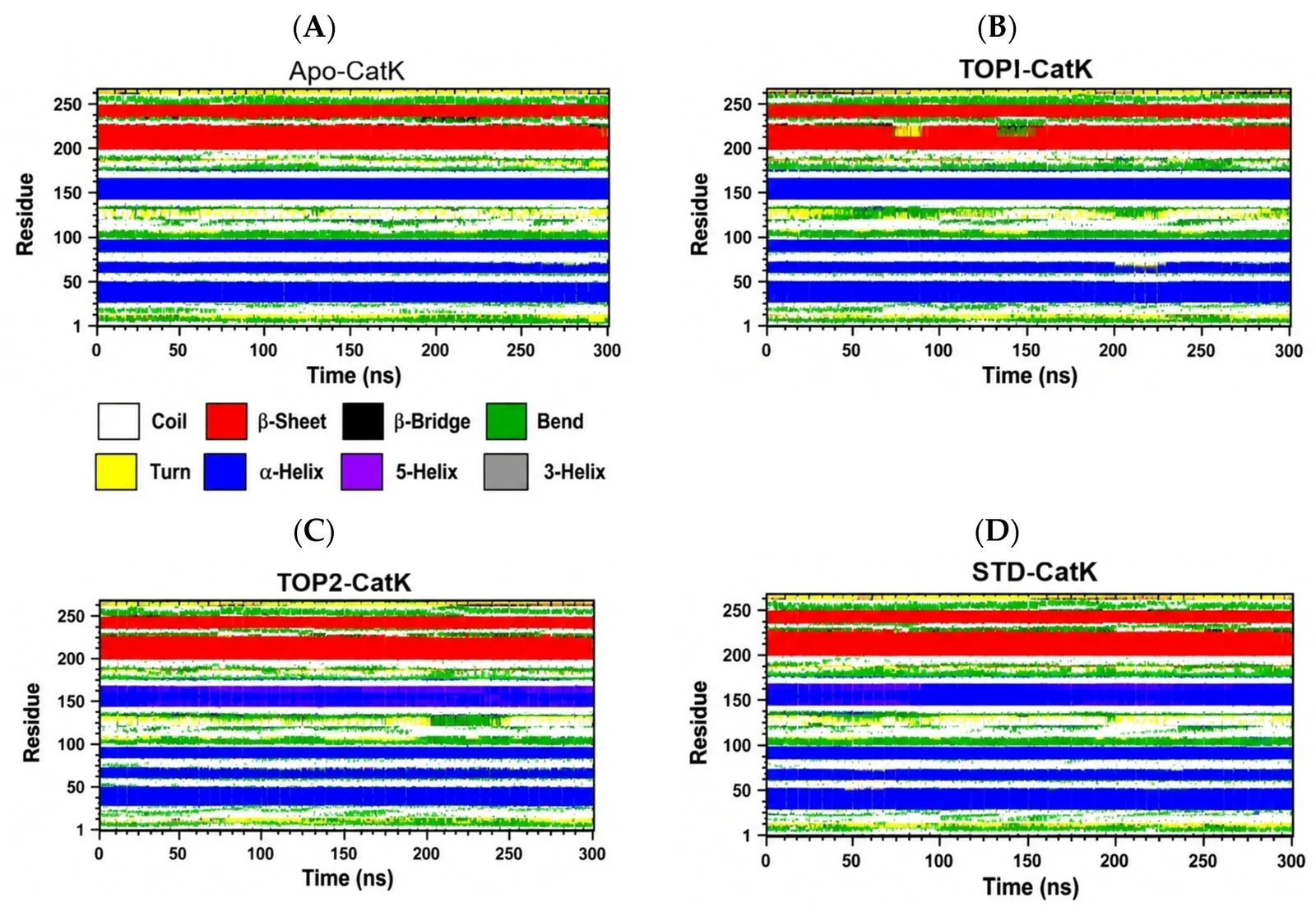
Secondary structure analysis of apo CatK and protein–ligand complexes during the 300 ns molecular dynamics simulation. The temporal evolution of DSSP-assigned secondary structure elements is shown for (**A**) apo CatK, (**B**) TOP1-CatK, (**C**) TOP2-CatK, and (**D**) STD-CatK. Secondary structure elements are color-coded according to the DSSP classification, including alpha helices, beta sheets, beta bridges, turns, bends, and coils.

In contrast, although most of the α-helices and β-sheets were preserved in the reference STD, the occurrence of diffused green and white regions illustrates mild flexibility in the loop regions, which suggests that STD binding enables the protein to maintain a major portion of its structure in the folded state, accompanied by local structural flexibility in the loop or coil regions. Variations in the loop regions are highlighted in the structural snapshots shown in Figure 10. The structural snapshots, taken at 25 ns intervals for both the ligand-bound and Apo forms, are shown, with 0, 100, and 200 ns selected to elucidate any differences observed in the flexible loop regions. The residue values from 80 to 105 were labeled as R1, and the residue values from 145 to 160 ns and 197 to 209 ns were designated as R2. The motion of these loop regions was then observed.

The results reveal that both regions, R1 and R2, tend to remain significantly unchanged, whereas in TOP2, the R1 region showed some flexibility, as indicated by the RMSF values. Although the R1 region remained flexible, it neither induced any structural distortion to the secondary structures nor affected the TOP2 binding to the active site. These results reveal that substantial changes in the secondary structure have not occurred in both the ligand-bound complexes, while the minor conformational alterations that were undergone due to the local movement of the loop regions upon TOP2 binding have also not induced any drastic effects in maintaining the native conformation over the entire 300 ns time period.

#### 2.3.8. Dynamic Cross-Correlation Matrix Analysis

**Figure 11:**
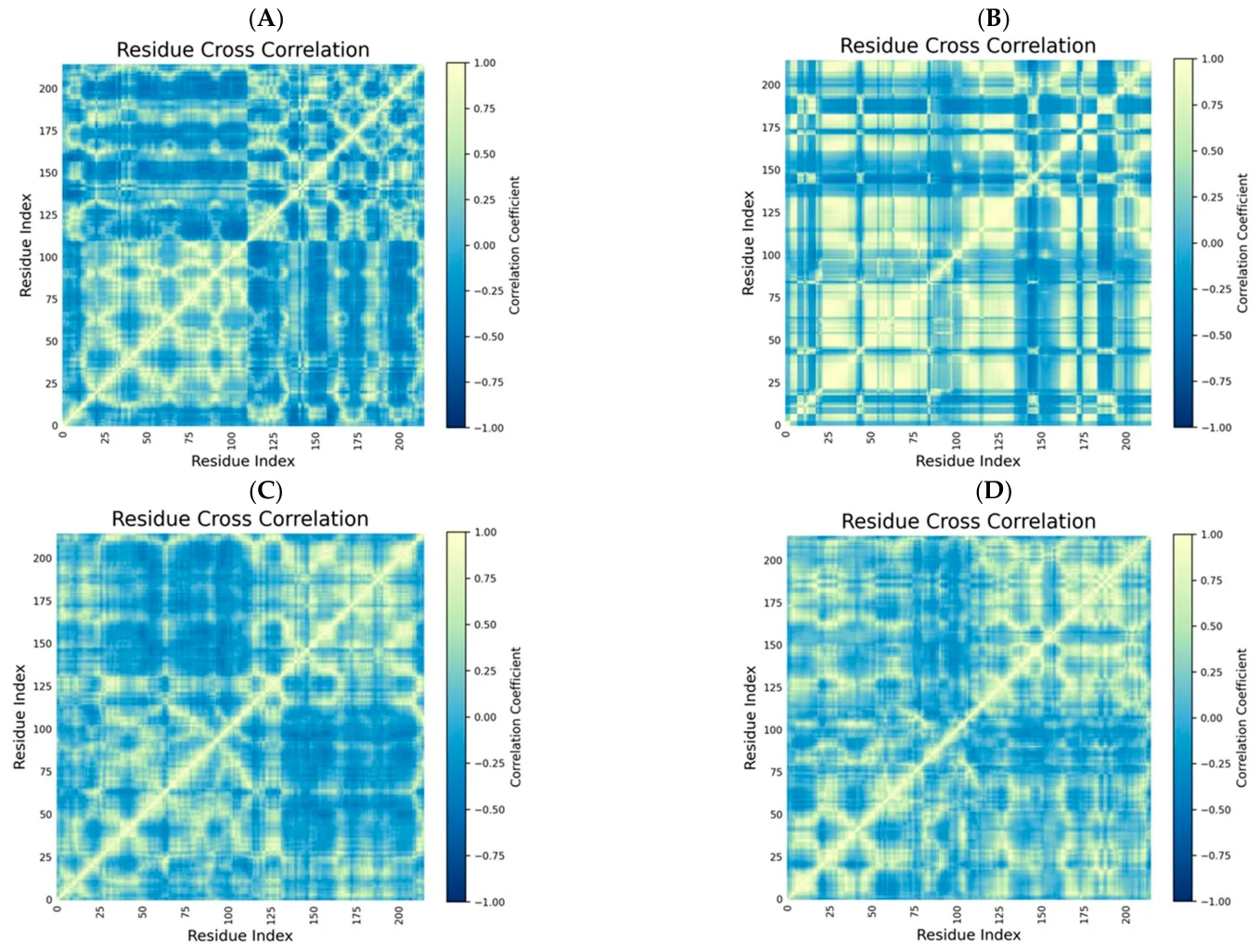
Dynamic cross-correlation matrix (DCCM) analysis of residue motions in the apo CatK domain and protein–ligand complexes during the 300 ns molecular dynamics simulation. Correlation maps were generated from the atomic fluctuations observed throughout the simulation. (**A**) Apo CatK, (**B**) TOP1 complex, (**C**) TOP2 complex, and (**D**) STD complex. Positive correlation values indicate coordinated residue motions, whereas negative correlation values represent anti-correlated movements between residue pairs.

Dynamic cross-correlation matrix (DCCM) analysis provides information about the coordinated atomic movements of each amino acid residue relative to the others during the MDS. Accordingly, DCCM was used to analyze the synchronous motion of Apo, TOP1, TOP2, and STD that were bound to the CatK protein to assess the impact of each bound ligand. As shown in Figure 11, the modest appearance of the yellow and blue regions indicates fewer opposite and non-correlated movements of the residues. On the other hand, in the TOP1-bound systems, prominent distinct yellow color patterns with fewer blue regions were observed than were recorded in the Apo system. This shows that robust strong positive correlations among the residues were confirmed, indicating that TOP1 binding influenced coordinated residue movements and the protein’s synchronized dynamics throughout the simulation process. With respect to the TOP2, sharp diagonal and localized correlations can be seen. Furthermore, the moderate presence of blue indicates that the bound TOP2 constrained overall motion while allowing localized changes, thereby confirming stabilization of the protein’s conformational state. Accordingly, in the reference STD-bound system, a greater proportion of heterogeneous patterns was observed. As has been observed, a combination of many smaller yellow and blue areas was present, suggesting that correlated movements are discontinuous, and that STD binding leads to fewer synchronized atomic motions with greater heterogeneity.

#### 2.3.9. MM-PBSA Analysis

**Figure 12:**
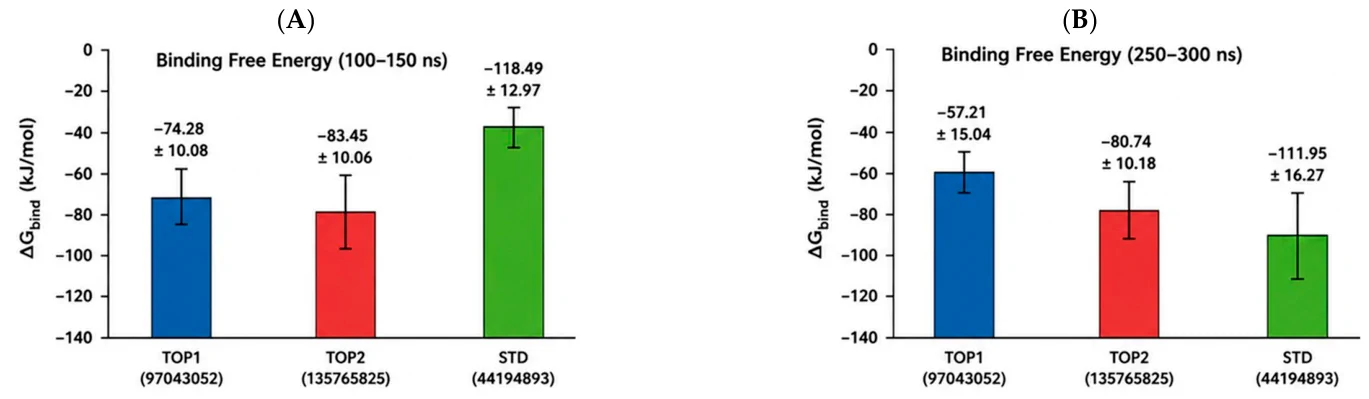

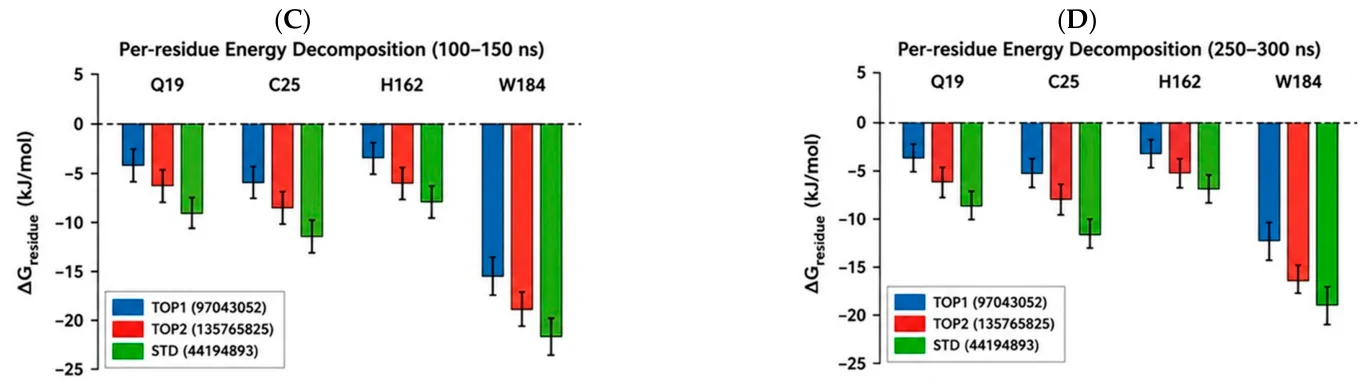
Binding free energy estimation and active site residue energy decomposition of CatK ligand complexes determined by molecular mechanics Poisson–Boltzmann surface area (MM-PBSA (MM/PBSA) analysis. (**A**) Average binding free energy (ΔG_binding_) calculated from trajectory frames extracted between 100 and 150 ns. (**B**) ΔG_binding_ calculated from trajectory frames extracted between 250 and 300 ns. (**C**) Per-residue free energy decomposition of key active site residues (Q19, C25, H162, and W184) during the 100–150 ns interval. (**D**) Per-residue free energy decomposition of the same residues during the 250–300 ns interval. Error bars represent standard deviations calculated from the analyzed trajectory frames.

MM/PBSA analysis was used as an approximate post-MD method to estimate relative binding free energy TOP1, TOP2, and STD with CatK. Mean ∆G_binding_ values were calculated for two trajectory intervals, 100–150 and 250–300 ns, as shown in Figure 12.

The mean Δ_Gbinding_ values for TOP1-CatK were −74.28 ± 10.08 kJ/mol at 100–150 ns and −57.21 ± 15.04 kJ/mol at 250–300 ns; corresponding values for TOP2-CatK were −83.45 ± 10.06 and −80.74 ± 10.18 kJ/mol, respectively, whereas STD-CatK showed −118.49 ± 12.97 and −111.95 ± 16.27 kJ/mol. Thus, TOP2 had more negative, and therefore more favorable, MM/PBSA binding free energies than TOP1 at both analyzed intervals, while STD had the most negative values overall. The individual van der Waals, electrostatic, polar solvation, and nonpolar solvation contributions are summarized in Table 4.

**Figure 13:**
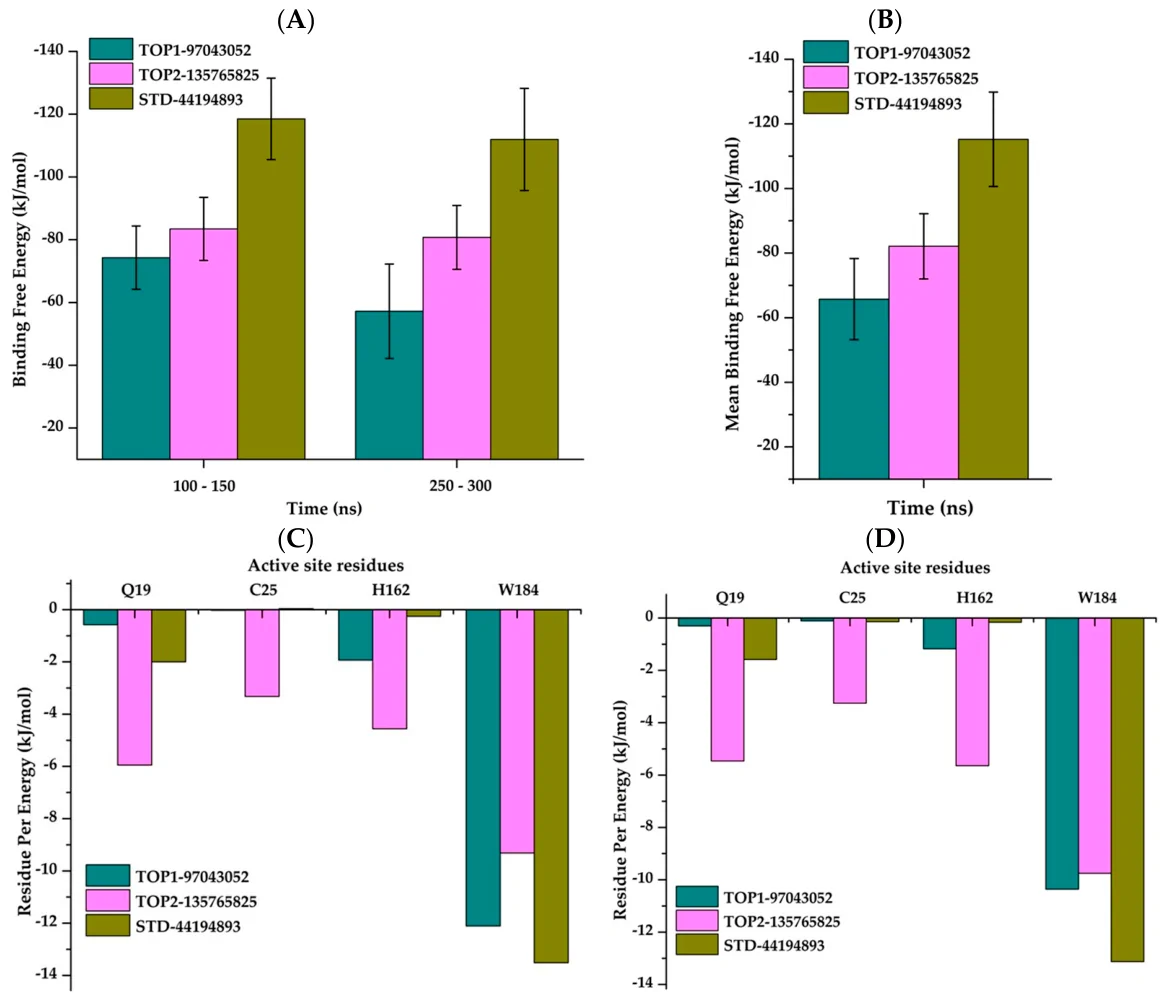
Comparative visualization of MM/PBSA binding energetics and active site residue energy decomposition for CatK ligand complexes. (**A**) Mean ΔGbinding values at 100–150 ns. (**B**) Mean ΔGbinding values at 250–300 ns. More negative values indicate more favorable predicted binding energetics. (**C**) Per-residue energy contributions of Q19, C25, H162, and W184 at 100–150 ns. (**D**) Corresponding per-residue contributions at 250–300 ns. Error bars represent SD across analyzed trajectory frames. These residue-level values are MM/PBSA decomposition terms and are not independent experimental measures of residue-specific affinity.

To facilitate comparison of the MM/PBSA results, Figure 13 summarizes the overall binding free energies and the energetic contributions of selected CatK active site residues. Consistent with the quantitative results presented in Figure 12, TOP2 showed more negative binding free energies than TOP1 at both analyzed trajectory intervals, whereas STD exhibited the most favorable overall binding energetics (Figure 13A,B). Per-residue decomposition further showed that Q19, C25, H162, and W184 contributed differently among the complexes (Figure 13C,D). W184 provided a prominent favorable contribution, while TOP2 showed favorable contributions distributed across multiple active site residues, including Q19, C25, H162, and W184.

These residue-level patterns support the overall energetic distinction between TOP1 and TOP2 but should be interpreted as MM/PBSA-derived energetic contributions rather than independent measures of residue-specific binding affinity. Thus, Figure 13 provides a comparative visualization of the MM/PBSA results rather than an additional independent binding energy analysis.

## 3. Discussion

CatK is a lysosomal cysteine protease that is highly expressed in osteoclasts and plays a central role in the degradation of type I collagen, the major organic component of bone matrix. Dysregulated CatK activity contributes to excessive bone resorption and has been strongly associated with osteoporosis and other bone-related disorders [8,12]. Previous studies have shown that CatK inhibition can reduce bone resorption while relatively preserving bone formation, making CatK an attractive anti-osteoporotic target [10]. However, the clinical development of several synthetic CatK inhibitors, including odanacatib and balicatib, has been limited by safety concerns, off-target effects, and a lack of regulatory approval [12,19]. Therefore, the identification of safer and structurally diverse CatK inhibitors remains an important area of drug discovery.

Molecular docking has previously been applied to identify and characterize small-molecule inhibitors targeting CatK and to investigate their interactions with functionally relevant binding sites [20]. The molecular docking analysis has identified TOP1 and TOP2 as the highest ranked natural compounds, as they exhibited stronger predicted binding affinities than the reference inhibitor. Importantly, both compounds occupied the catalytic binding pocket of CatK and interacted with key residues involved in ligand recognition, including Q19 and W184, which contribute to substrate recognition and stabilization within the active site. TOP1 additionally established hydrogen bond interactions with N161, suggesting a more favorable binding orientation and stronger intermolecular interactions than TOP2. Previous structural and computational studies have demonstrated that hydrogen bond formation and hydrophobic interactions with catalytic site residues are major determinants of ligand affinity and specificity toward CatK, thereby enhancing inhibitory activity and complex stability [10,21,22]. Furthermore, several recent virtual screening studies have shown that compounds exhibiting multiple hydrogen bonds and extensive hydrophobic contacts generally display improved docking scores and are more likely to maintain stable binding during molecular dynamics simulations [23,24]. Although molecular docking provides only a static representation of protein–ligand interactions, it is widely accepted as an efficient first-step screening strategy for prioritizing compounds for more rigorous dynamic and free energy analyses. Therefore, TOP1 and TOP2 were selected for subsequent molecular dynamics simulations to evaluate the stability of their binding modes under physiologically relevant conditions.

TOP1 and TOP2 satisfied Lipinski’s rule of five, indicating that their basic physicochemical properties fall within commonly used drug-likeness boundaries [25,26]. This observation should not, however, be equated with favorable oral absorption. In Table 3, human intestinal absorption is predicted as negative for both compounds, and the Caco-2/MDCK outputs do not justify a definitive claim of efficient epithelial permeability. In addition, the CYP and P-glycoprotein predictions indicate compound-specific metabolic interaction liabilities, particularly for TOP2, while the numerical toxicity model outputs raise issues that require experimental clarification. Moreover, transporter-mediated disposition, particularly involving P-glycoprotein, together with membrane permeability, can substantially influence drug distribution and pharmacokinetic behavior [27]. The absence of predicted BBB permeability may limit central exposure, but this alone cannot establish a favorable safety profile. Accordingly, the ADMET results are interpreted as mixed and hypothesis-generating rather than evidence of advantageous pharmacokinetics or safety [28,29].

In this study, an integrated computational strategy combining virtual screening, molecular docking, ADMET prediction, molecular dynamics simulation, and MM/PBSA binding free energy analysis was used to identify potential CatK inhibitors from the MEGxM natural product database. Among the screened compounds, TOP1 and TOP2 showed higher predicted binding affinities than the reference ligand. The docking scores of TOP1 and TOP2 were −8.2 and −7.8 kcal/mol, respectively, as compared with −6.6 kcal/mol for the reference ligand. These values are comparable to those reported in previous computational studies of CatK inhibitors, in which natural compounds and phytochemicals, such as quercetin and related polyphenols, were predicted to interact favorably with CatK active site residues [30]. Recent deep learning and molecular docking studies have also identified quercetin, γ-linolenic acid, and benzyl isothiocyanate as potential CatK inhibitors, supporting the relevance of natural product-derived compounds in CatK-targeted osteoporosis research [30].

The predicted binding modes suggest that TOP1 and TOP2 interact with CatK residues implicated in ligand recognition and active site stabilization. In the present docking analysis, TOP1 formed hydrogen bonds with Q19 and N161 and additional contacts with W184 and C25, whereas TOP2 formed hydrogen bonds with C25, H162, Q21, and Q19 and an additional contact with W184 (Table 1). During MD, the complexes showed different interaction patterns rather than a single uniformly superior profile. TOP1 had slightly lower average RMSD/RMSF values, whereas TOP2 showed more favorable MM/PBSA binding energetics. These observations support considering both candidates for experimental testing, but they do not establish inhibitory potency or rank biochemical affinity.

Molecular dynamics simulations indicated that both TOP1-CatK and TOP2-CatK remained conformationally stable over 300 ns, with no major ligand-associated disruption of global CatK structure based on RMSD, RMSF, Rg, SASA, and hydrogen bond analyses [31,32]. MM/PBSA provided a complementary energetic estimate [33]. Importantly, TOP2 showed more favorable ΔGbinding values than TOP1 at both analyzed intervals, while STD showed the most favorable values overall. Thus, the MD and MM/PBSA results should be interpreted together: TOP1 showed slightly lower structural fluctuations, whereas TOP2 showed stronger predicted MM/PBSA binding energetics. Neither computational metric alone establishes experimental binding affinity or inhibition.

The contrasting TOP1 and TOP2 profiles illustrate why docking scores, trajectory stability, hydrogen bond counts, and MM/PBSA energies should not be collapsed into a single claim of superiority. TOP1 ranked first by docking score and showed slightly lower average structural fluctuations, whereas TOP2 had more negative MM/PBSA binding free energies at both analyzed windows. STD remained the most favorable system by MM/PBSA. Accordingly, TOP1 and TOP2 are treated as complementary computational candidates for subsequent validation rather than as confirmed inhibitors or as evidence that TOP1 is globally superior. Several limitations remain. This study is entirely computational and lacks enzymatic or cell-based validation; docking used a rigid receptor; MM/PBSA is approximate and does not fully capture entropic contributions; and ADMET/toxicity outputs are model predictions. Future work should verify CatK inhibition biochemically, quantify binding using appropriate biophysical assays, evaluate osteoclast-related activity, and experimentally characterize absorption, metabolism, and toxicity before in vivo testing.

## 4. Materials and Methods

### 4.1. Virtual Screening and Protein Preparation

Virtual screening of the compounds was performed using the MEGxM database, which contained approximately 6500 natural product-derived compounds. This work aimed to use this public database to identify potential compounds that could serve as CatK inhibitors. These compound structures were downloaded and saved in the Structure-Data File (SDF) format and subsequently subjected to energy minimization using the MMFF94 force field and the conjugate gradient algorithm prior to molecular docking [18,34,35]. Likewise, the CatK protein structure was retrieved from the Research Collaboratory for Structural Bioinformatics Protein Data Bank (RCSB PDB) under 2ATO [36]. All crystallographic water molecules were removed using a Biovia Discovery Studio Visualizer (Biovia, San Diego, CA, USA). Subsequently, hydrogen atoms were added, bond orders were assigned, missing side chains and atoms were repaired when necessary, and protonation states of ionizable residues were assigned according to the physiological pH (7.4).

### 4.2. Molecular Docking Analysis

Ligands from the MEGxM database were converted to 3D PDB format using Open Babel version 3.1.1 [37]. Molecular docking was performed with AutoDock 4.2 using the Lamarckian genetic algorithm [38]. The docking grid was centered on the 2ATO active site region (X = 30.7873, Y = 54.2514, Z = 64.0208), with 40 × 40 × 40 grid dimensions and 0.5 Å spacing. Kollman charges were assigned to the protein and Gasteiger charges to ligands. Docking parameters included a population size of 150, 2,500,000 energy evaluations, a mutation rate of 0.02, a crossover rate of 0.8, and 10 independent runs per ligand; the lowest energy pose was selected for analysis. Ligand rotatable bonds were flexible and the receptor was treated as rigid. TOP-ranked poses were inspected using PyMOL 2.5 and BIOVIA Discovery Studio Visualizer 2021. STD (PubChem ID: 44194893) was retained as a comparative reference ligand in the docking/MD analyses. Docking protocol validation by crystallographic ligand redocking is described separately in Section 4.3 and should not be conflated with the comparative STD ligand.

### 4.3. Docking Protocol Validation

The CatK structure (PDB ID: 2ATO) was used as the receptor structure for molecular docking. The crystallographic ligand present in the 2ATO structure was retained as a structural reference for comparison but was not used as the docking validation ligand. A structurally distinct reference compound (STD, PubChem ID: 44194893) was independently docked into the CatK binding pocket using the same docking protocol applied to TOP1 and TOP2. Following docking, the docked STD pose was structurally superimposed onto the crystallographic ligand conformation present in the 2ATO structure using PyMOL. The RMSD between the crystallographic ligand and the docked STD pose was calculated to assess the similarity of their binding poses within the CatK binding pocket (Figure S1). The calculated RMSD was 0.663 Å. This analysis was used as a comparative structural assessment of the predicted binding pose of STD relative to the experimentally observed ligand conformation and was not considered a conventional crystallographic ligand redocking validation.

### 4.4. Ligand Parameter Generation

Ligand topology and force field parameters were generated using the CHARMM General Force Field (CGenFF) server [39,40]. The generated topology and parameter files were incorporated into the CHARMM36 force field for subsequent molecular dynamics simulations.

### 4.5. Drug-likeness and Pharmacokinetic Properties Prediction

Physicochemical and drug-likeness properties of TOP1 and TOP2 were evaluated using SwissADME web server (Swiss Institute of Bioinformatics, Lausanne, Switzerland; accessed on 12 June 2026 [41], including molecular weight, hydrogen bond acceptors/donors, cLogP, TPSA, and Lipinski’s rule of five [42]. ADMET predictions were obtained using the platform reported in this study for absorption, distribution, CYP metabolism, and excretion endpoints [43], while toxicity-related predictions were obtained using ProTox 3.0 web server (Charité–Universitätsmedizin Berlin, Berlin, Germany; accessed on [actual date of analysis]) [44]. Canonical SMILES retrieved from PubChem were used as input. Lipinski compliance was interpreted only as a drug-likeness descriptor and not as proof of oral bioavailability. Model outputs were reported as predictions and were not converted into experimental toxic/non-toxic classifications without an explicit model-defined threshold.

### 4.6. Molecular Dynamics Simulation

Molecular dynamics simulations were performed to evaluate the structural stability and dynamic behavior of the CatK ligand complexes [45]. The two highest ranked compounds, TOP1 and TOP2, the reference ligand STD, and the ligand-free apo CatK were selected for MD simulations using the GROMACS software package (version 2019.4) with the CHARMM36 force field [42,46]. Ligand topology files were generated using the CGenFF server [39,40]. Initially, each system was subjected to energy minimization using the steepest descent algorithm until the maximum force was below 1000 kJ mol^−1^ nm^−1^. The minimized complexes were subsequently solvated in a cubic simulation box containing the simple point charge (SPC) explicit water model, and periodic boundary conditions were applied in all three dimensions. Counterions (Na^+^ and Cl^−^) were added using the gmx genion module to neutralize the system and achieve a physiological salt concentration of 0.15 M. Following energy minimization, the systems were equilibrated in two sequential phases. First, analysis involving a constant number of particles, volume, and temperature (NVT) ensemble was performed for 100 ps at 310 K using the modified Berendsen (V-rescale) thermostat to stabilize the system temperature. Subsequently, a constant number of particles, combined with a constant pressure and temperature (NPT) ensemble, was conducted for 100 ps at 310 K and 1 bar using the Parrinello–Rahman barostat to stabilize system pressure and density while maintaining periodic boundary conditions. Temperature coupling was performed using the V-rescale thermostat with a coupling constant (τT) of 0.1 ps, whereas pressure coupling employed the Parrinello–Rahman barostat with a coupling τP of 2.0 ps [47,48].

After equilibration, production MD simulations were carried out for 300 ns under periodic boundary conditions with a time step of 2 fs, while trajectory coordinates were recorded every 10 ps for subsequent analyses. The structural stability of each system was evaluated by calculating the RMSD, RMSF, Rg, SASA, hydrogen bond occupancy, PC, FEL, and secondary structure evolution using the built-in GROMACS analysis tools. The binding free energy values of the CatK ligand complexes were estimated using the MM/PBSA approach implemented in the g_mmpbsa package. ΔG_binding_ values were then calculated according to the following Equation (1):

(1) ΔG_binding_ = G_complex_ − (G_protein_ + G_ligand_)

where G_complex_, G_protein_, and G_ligand_ represent the free energy values of the protein–ligand complex, protein, and ligand, respectively. The free energy value of each molecular state (Gx) was estimated using Equation (2):

(2) Gx = (EMM) − TS + (G_solvation_)

where EMM determines the average molecular mechanics, TS refers to the entropic contribution, and Gsolvation represents the total solvation free energy of the protein bound with the ligand, consisting of polar (Poisson–Boltzmann), polar state (Gpolar), and nonpolar state (Gnonpolar) contributions. The EMM was calculated by considering the electrostatic and vdW interactions between the protein–ligand complex, as shown in Equations (3) and (4). All covalent bonds involving hydrogen atoms were constrained using the LINCS algorithm [18]. Long-range electrostatic interactions were treated using the particle mesh Ewald (PME) method with a Fourier spacing of 0.12 nm [49,50]. A short-range cutoff of 1.2 nm was applied to both electrostatic and vdW interactions. The nonpolar solvation energy was estimated from the SASA.

(3) EMM = E_bonded_ + E_non–bonded_

(4) Gsolvation = Gnonpolar + Gpolar

To quantify the energy contributions of the desired active site-interacting amino acids in the putative agonists, per-residue free energy decomposition was performed to identify the contributions of amino acid residues located within 4 Å of the ligand-binding site to the overall binding affinity, using snapshots extracted from the equilibrated final 50 ns of the simulation trajectory. Residues within 4 Å of the ligand throughout the equilibrated trajectory were included in the per-residue energy decomposition analysis. These amino acids were considered, and the g_mmpbsa tool was employed to predict the binding energy capabilities from the MM-PBSA run [50]. Residue-wise binding free energies were subsequently calculated using the MmPbsaDecomp.py script, which excluded the estimation of entropy (TΔS), while the predicted free energies denote only enthalpic parameters (molecular mechanics and solvation energies) [50]. The final MM-PBSA calculations were individually performed using trajectories extracted at two different simulation windows (100–150 and 250–300 ns). These two intervals were chosen to predict the binding free energy values across the various simulation time period. Binding free energy values were then calculated as the average of all selected frames and reported as mean ± standard deviation (SD) values.

## 5. Conclusions

Cathepsin K remains an attractive therapeutic target for osteoporosis because of its central role in osteoclastic bone resorption. The integrated computational workflow prioritized TOP1 and TOP2 as natural product-derived CatK-binding candidates with complementary profiles. TOP1 ranked more favorably by docking and showed slightly lower average structural fluctuations, whereas TOP2 exhibited more favorable MM/PBSA binding free energies at both analyzed trajectory intervals; STD showed the most favorable MM/PBSA energetics overall. Both TOP1 and TOP2 satisfied Lipinski’s rule of five, but their predicted ADMET profiles were mixed and included negative human intestinal absorption predictions and potential metabolic/toxicity liabilities. Therefore, neither compound can presently be described as a confirmed CatK inhibitor, an orally bioavailable lead, or a validated therapeutic candidate. Biochemical CatK inhibition assays, direct binding measurements, cellular osteoclast studies, and experimental pharmacokinetic/toxicological evaluation are required before therapeutic potential can be established.

## Figures and Tables

**Table 1:** Molecular docking analysis of the reference ligand (STD) and the top 5 similarity-selected compounds (TOP1–TOP5) against the target protein. Docking scores, numbers of hydrogen bonds, interacting residues involved in hydrogen bonding, numbers of other non-covalent interactions, and corresponding interacting residues within the binding pocket are presented.

Ligand	PubChem ID	Docking Score (kcal/mol)	H-Bonds (Number)	H-Bond Residues	Other Non-Covalent Interactions (Number)	Other Interacting Residues
STD	44194893	−6.6	2	W184	1	C22
TOP1	97043052	−8.2	2	Q19, N161	2	W184, C25
TOP2	135765825	−7.8	4	C25, H162, Q21, Q19	1	W184
TOP3	137955144	−7.8	2	Q19, Q21	0	-
TOP4	102571585	−7.7	5	Q21, C22, Q19, W184, H162	1	W184
TOP5	92290113	−7.7	2	Q143, H162	1	W183

**Table 2:** Drug-likeness properties of the top-ranked compounds identified by molecular docking.

Ligand (PubChem ID)	cLog P	MW (g/mol)	TPSA	HA (*n*)	HD (*n*)	ROT (*n*)	Lipinski Violation
TOP1 (97043052)	1.653	432.14	104.97	9.0	1.0	1.0	No
TOP2 (135765825)	2.61	355.1	84.22	6.0	2.0	0.0	No

Abbreviations: cLogP, calculated octanol/water partition coefficient; HA, H-bond acceptor; HD, H-bond donor; MW, molecular weight; *n*, number; TPSA, topological polar surface area.

**Table 3:** Predicted absorption, distribution, metabolism, excretion, and toxicity (ADMET) properties of the top-ranked compounds TOP1 and TOP2.

Property	TOP1 (PubChem ID: 97043052)	TOP2 (PubChem ID: 135765825)
**Absorption**		
Caco2 cell permeaboolity	−5.489	−4.892
MDCK permeability	0.0	0.0
P-gp1	negative	negative
Human intestinal absorption	negative	negative
P-glycoprotein I inhibitor	negative	+++
**Distribution**		
BBB permeability	negative	negative
**Metabolism**		
CYP2D6 substrate	negative	++
CYP3A4 substrate	+++	+
CYP1A2 inhibitor	negative	+++
CYP2C19 inhibitor	negative	negative
CYP2C9 inhibitor	negative	negative
CYP2D6 inhibitor	negative	negative
CYP3A4 inhibitor	negative	+
**Excretion**		
Total clearance (mL/min/kg)	5.04	1.43
**Toxicity**		
AMES toxicity	0.94	0.84
Genotoxicity	1.00	0.98
ORAT (LD_50_)	0.41	0.40
Hepatotoxicity	0.89	0.82
Carcinogenicity	0.50	0.68
HEK293 cytotoxicity	0.68	0.74

Abbreviations:BBB, blood–brain barrier; Caco2, human colorectal adenocarcinoma cell line; CYP, cytochrome P450; HEK293, human embryonic kidney 293 cells; LD_50_, median lethal dose; MDCK, Madin–Darby canine kidney; P-gp1, P-glycoprotein 1. For categorical prediction outputs, negative indicates a negative prediction, whereas +, ++, and +++ indicate increasing levels of positive prediction confidence/probability, as reported by the prediction platform.

**Table 4:** MM/PBSA binding free energy components of TOP1–CatK, TOP2–CatK, and reference ligand (STD)–CatK complexes calculated over the 100–150 ns and 250–300 ns molecular dynamics simulation intervals.

System	van der Waal Energy (kJ/mol)	Electrostatic Energy (kJ/mol)	Polar Solvation Energy (kJ/mol)	SASA Energy (kJ/mol)
TOP1 (100–150 ns)	−141.31 ± 9.82	−4.34 ± 7.96	84.30 ± 10.98	−12.93 ± 1.10
TOP1 (250–300 ns)	−121.58 ± 13.43	−12.42 ± 10.35	89.28 ± 10.85	−12.49 ± 1.23
TOP2 (100–150 ns)	−136.61 ± 10.31	−56.75 ± 10.61	121.82 ± 8.46	−11.92 ± 0.70
TOP2 (250–300 ns)	−132.40 ± 9.65	−45.31 ± 12.18	109.17 ± 12.36	−12.20 ± 0.81
STD (100–150 ns)	−192.56 ± 9.93	−23.93 ± 5.19	114.94 ± 10.34	−16.94 ± 1.07
STD (250–300 ns)	−177.87 ± 21.25	−23.65 ± 13.85	105.91 ± 23.21	−16.33 ± 1.87

## Data Availability

The original contributions presented in this study are included in the article/Supplementary Materials. Further inquiries can be directed to the corresponding authors.

## Supplementary Materials

The following supporting information can be downloaded at: https://www.mdpi.com/article/10.3390/ijms27188258/s1.

## Abbreviations

The following abbreviations are used in this manuscript:

BBB	Blood–brain barrier
BMD	Bone mineral density
Caco-2	Human colorectal adenocarcinoma cell line
CatK	Cathepsin K
CGenFF	CHARMM General Force Field
cLogP	Calculated octanol/water partition coefficient
CYP	Cytochrome P450
DCCM	Dynamic cross-correlation matrix
DSSP	Define secondary structure protein
FEL	Free energy landscape
GROMACS	Groningen machine for chemical simulations
(Gx)	Free energy of each molecular state
HA	H-bond acceptors
HD	H-bond donors
HEK293	Human embryonic kidney 293 cells
KDE	Kernel density distributions
LD_50_	Median lethal dose
MD	Molecular dynamics
MDCK	Madin–Darby canine kidney
MEGxM	Medicinal and Economic Plants Genome Database
MM/PBSA	Molecular mechanics/Poisson–Boltzmann surface area
MW	Molecular weight
TOP2T	Number of particles, pressure, and temperature
NVT	Number of particles, volume, and temperature
OPG	Osteoprotegerin
PC	Principal component
P-gp1	P-glycoprotein 1
PME	Particle mesh Ewald
RANK	Receptor activator of nuclear factor kappa B
RANKL	Receptor activator of nuclear factor kappa B ligand
Rg	Radius of gyration
RMSD	Root mean square deviation
RMSF	Root mean square fluctuation
SASA	Solvent-accessible surface area
SMILES	Simplified molecular-iTOP2ut line entry systems
SPC	Simple point charge
TPSA	Topological polar surface area
TS	Entropic contribution
vdW	van der Waals
2D	Two-dimensional
3D	Three-dimensional
∆G_binding_	Binding free energy
τT	Coupling constant
