# Supplementary material for: Virtual Screening and Molecular Dynamics Simulations Identifying Natural Product-Derived Cathepsin K Inhibitors as Potential Therapeutics for Osteoporosis
Source: Int J Mol Sci. 2026 Sep 16;27(18):8258. doi: 10.3390/ijms27188258 (PMC13607286; doi:10.3390/ijms27188258)
Supplement: Supplementary file 1 [file ijms-27-08258-s001.zip › ijms-4453607-supplementary.pdf]

Supplementary Materials

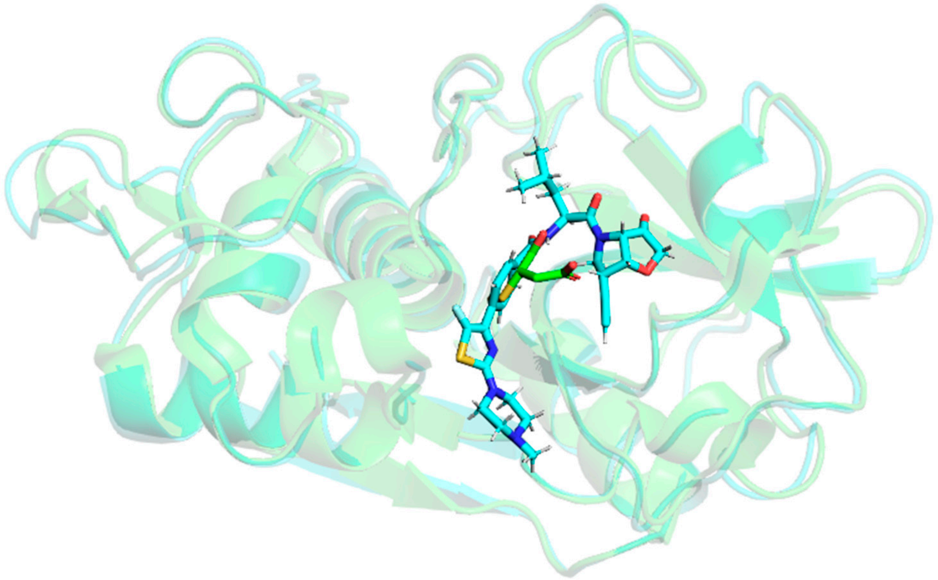

Figure S1. Structural superposition of the crystallographic and docked reference compound STD in the cathepsin K (CatK or 2ATO) binding site The crystallographic ligand present in the 2ATO structure is shown in green, whereas the independently docked reference compound STD (PubChem ID: 44194893) is shown in cyan. The docked STD pose was structurally superimposed onto the crystallographic ligand conformation within the CatK binding pocket. The RMSD between the crystallographic ligand and docked STD pose was 0.663 Å, indicating a high degree of similarity between the two ligand conformations.

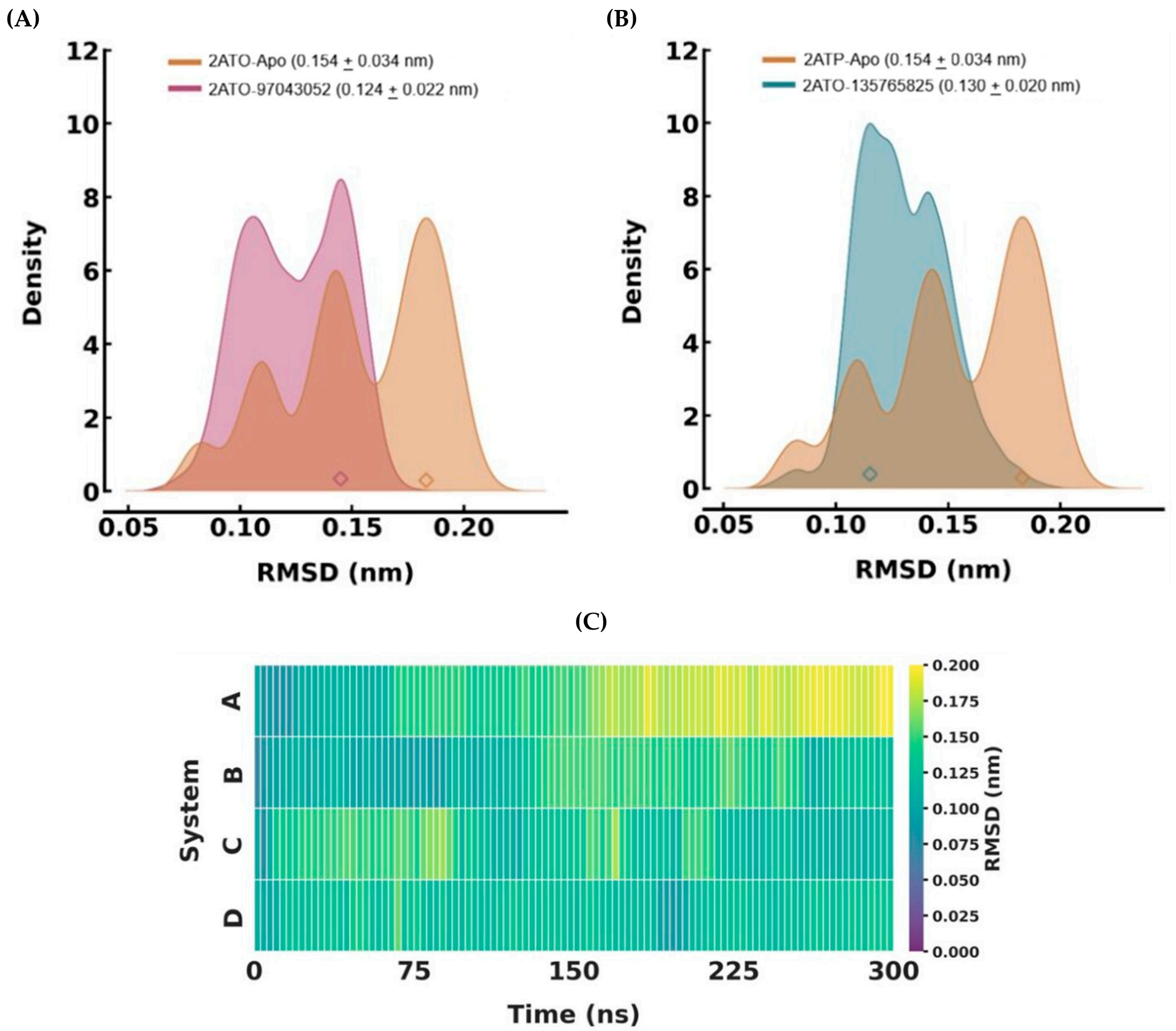

Figure S2. Probability density distribution and temporal evolution of root mean square deviation (RMSD) during 300 ns molecular dynamics simulations of the CatK (2ATO) systems. Kernel density estimation (KDE) plots showing the probability distribution of backbone RMSD values for the apo CatK (2ATO) and the ligand-bound complexes: **(A)** apo CatK (orange) and the TOP1 (PubChem ID: 97043052))–CatK complex (pink). **(B)** apo CatK (orange) and the TOP2 (PubChem ID: 135765825)–CatK complex (cyan). **(C)** Heatmap illustrating the temporal evolution of backbone RMSD values for four simulated systems over the 300 ns simulation period, with warmer colors (yellow) indicating higher RMSD values and cooler colors (blue-green) indicating lower RMSD values. The KDE distributions represent the probability of observing specific RMSD values throughout the simulations. The values in parentheses indicate the mean RMSD ± standard deviation (SD).

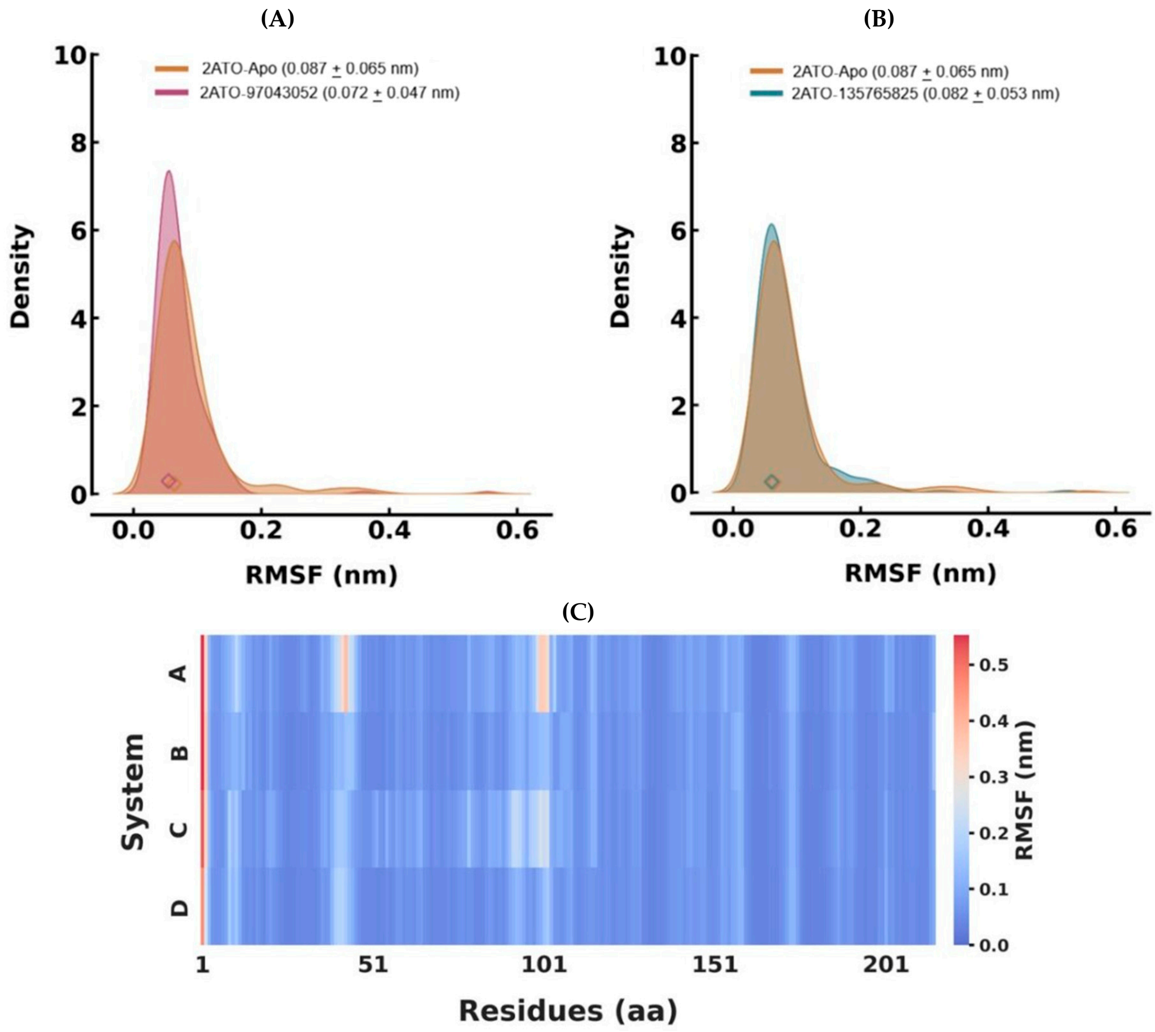

Figure S3. Distribution and residue-wise variation of root mean square fluctuation (RMSF) values of cathepsin K during the 300 ns molecular dynamics simulation. **(A)** KDE plot showing the distribution of RMSF values for apo CatK (2ATO) (orange) and the TOP1–CatK complex (pink). **(B)** RMSF density distribution of the apo protein and TOP2–CatK complex (cyan). **(C)** RMSF heatmap illustrating residue-wise backbone fluctuations for the four simulated systems during the 300 ns simulations, with warmer colors (yellow) indicating higher RMSF values and cooler colors (blue-green) indicating lower RMSF values. The KDE distributions represent the probability of observing specific RMSF values across the analyzed residdues. Values in parentheses indicate the mean RMSF ± SD.

(A)

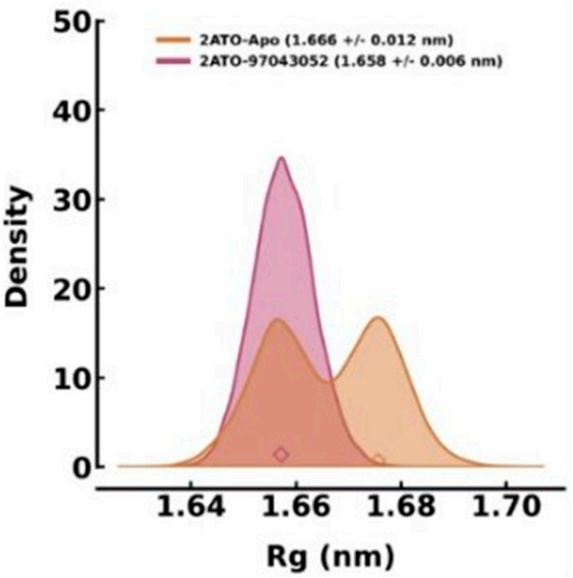

(B)

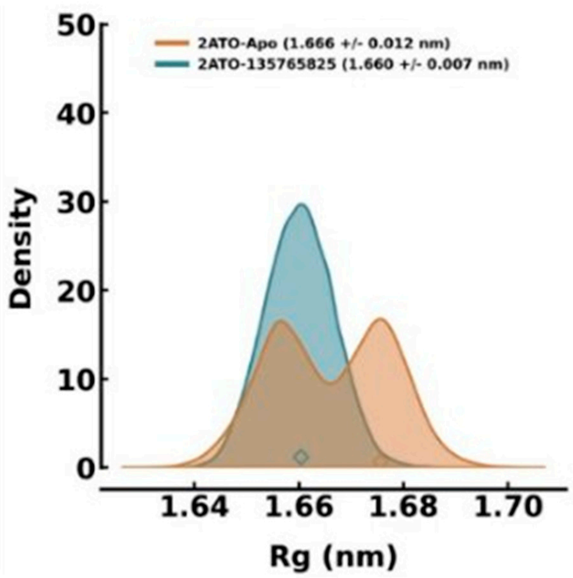

(C)

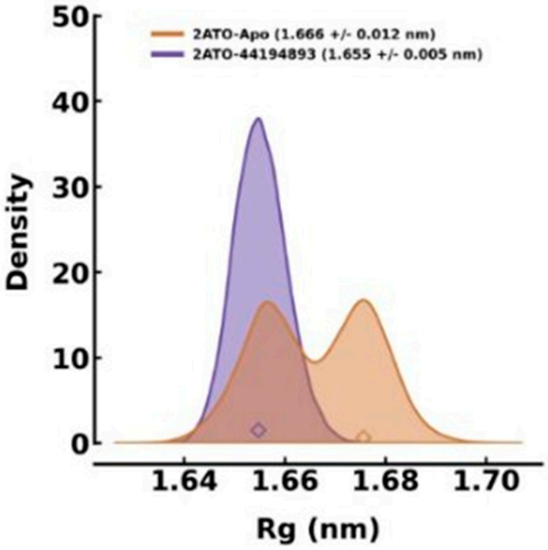

Figure S4. Probability density distribution of the radius of gyration (Rg) for cathepsin K during the 300 ns molecular dynamics simulation. KDE plots showing the distributions of Rg values for apo CatK (2ATO) and the ligand-bound complexes: **(A)** Apo CatK (orange) and the TOP1 (pink). **(B)** Apo CatK (orange) and the TOP2–CatK complex (cyan). **(C)** Apo CatK (orange) and the reference ligand (STD: (PubChem ID: 44194893)–CatK complex (purple). The Rg values provide a measure of the overall compactness of the protein structure throughout the simulation. Values in parentheses indicate the mean Rg ± SD.

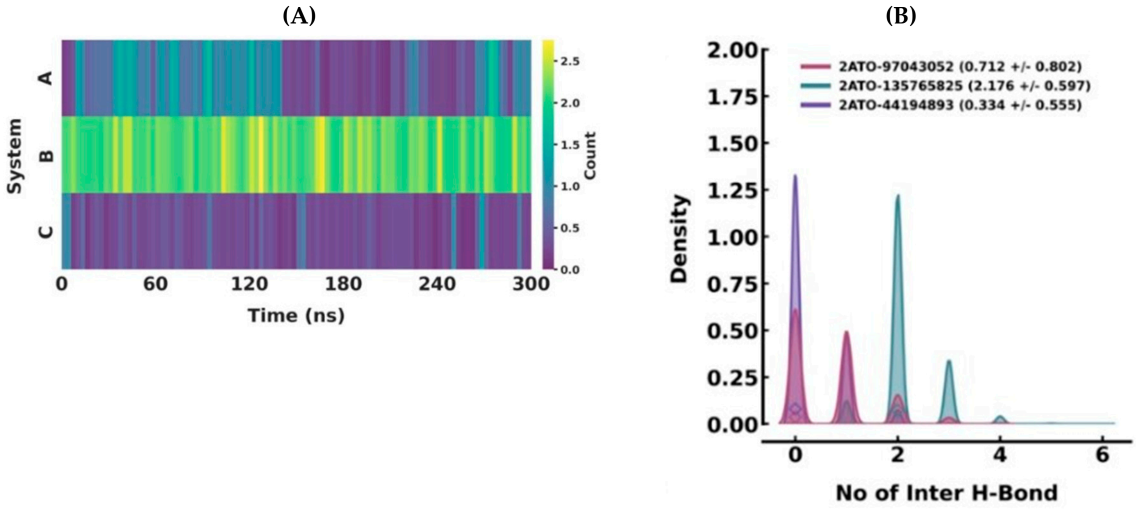

Figure S5. Hydrogen bond occupancy and probability distribution of intermolecular hydrogen bonds during the 300 ns molecular dynamics simulations. **(A)** Heatmap illustrating the temporal evolution of the number of intermolecular hydrogen bonds formed between CatK (2ATO) and the respective ligands throughout the 300 n simulation. Color intensity represents the hydrogen bond count, with warmer colors indicating a greater number of hydrogen bonds. **(B)** KDE plots showing the probability distribution of the numbers of intermolecular hydrogen bonds for the TOP1–CatK, TOP2–CatK, and reference ligand (STD)–CatK complexes. Values in parentheses represent the mean number of hydrogen bonds ± SD.

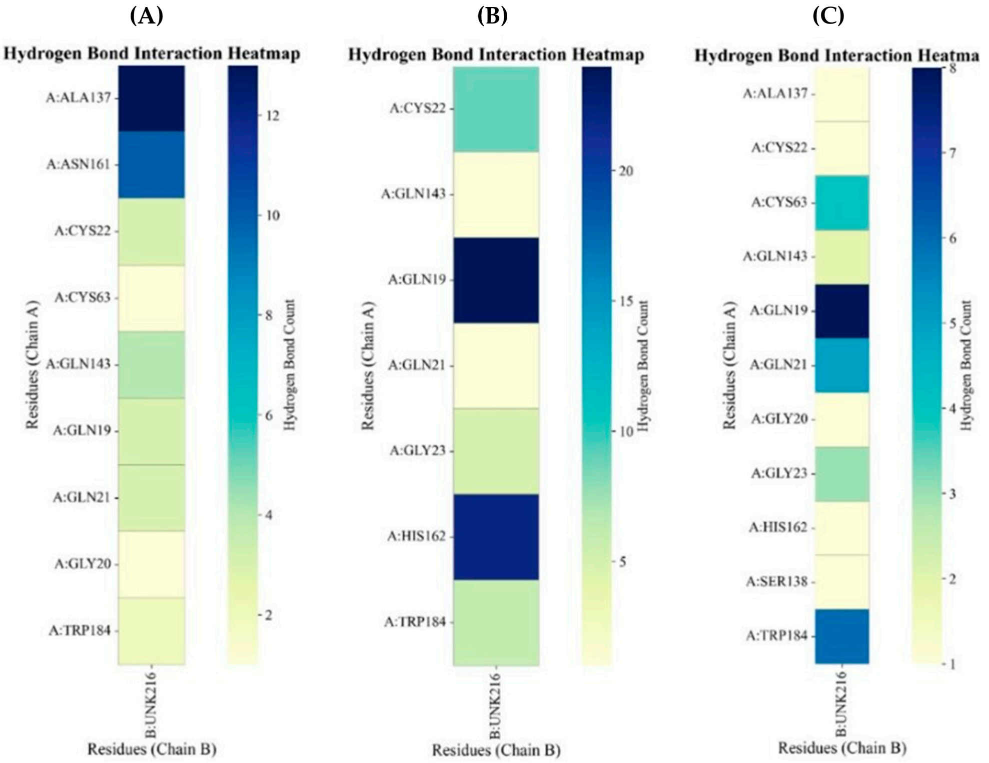

Figure S6. Residue-wise hydrogen bond interaction heatmaps of CatK–ligand complexes during the 300 ns molecular dynamics simulations. Heatmaps showing the frequency of hydrogen bond formation between CatK (2ATO) active-site residues and the respective ligands throughout the simulations. Color intensity represents hydrogen bond occupancy, with darker shades indicating more frequent hydrogen bond formation. **(A)** TOP1–CatK complex. **(B)** TOP2–CatK complex. **(C)** Reference ligand (STD)–CatK complex.

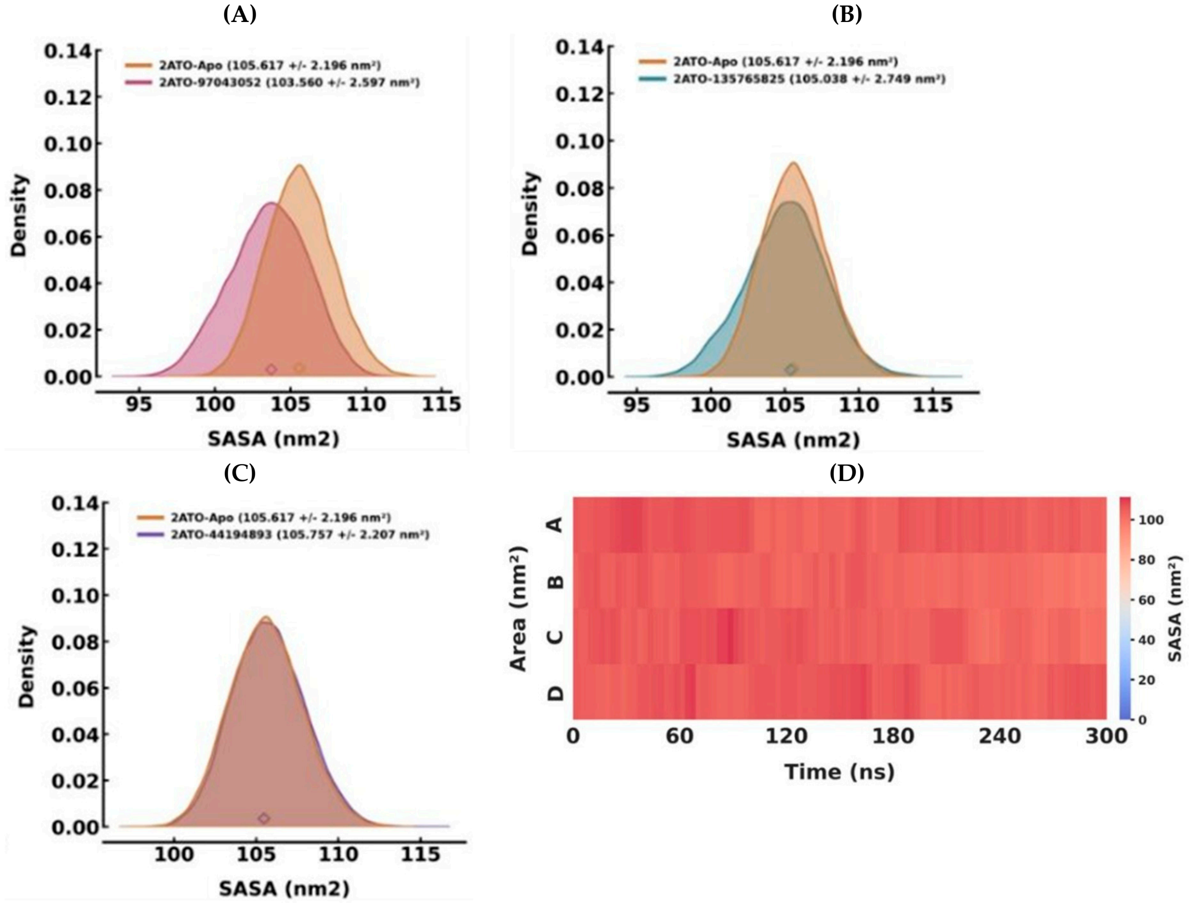

Figure S7. Probability density distribution and temporal evolution of solvent-accessible surface area (SASA) during the 300 ns molecular dynamics simulations of the CatK systems. **(A-C)** KDE plots showing the probability distributions of SASA values for apo CatK and the the ligand-bound complexes: (A) Apo CatK (orange) and the TOP1–CatK complex (pink); **(B)** Apo CatK (orange) and the TOP2–CatK complex (cyan); and **(C)** Apo CatK (orange) and the reference ligand (STD)–CatK complex (purple). **(D)** Heatmap showing the temporal evolution of SASA values for the four simulated systems throughout the 300 ns simulations. Values in parentheses indicate mean SASA ± SD.

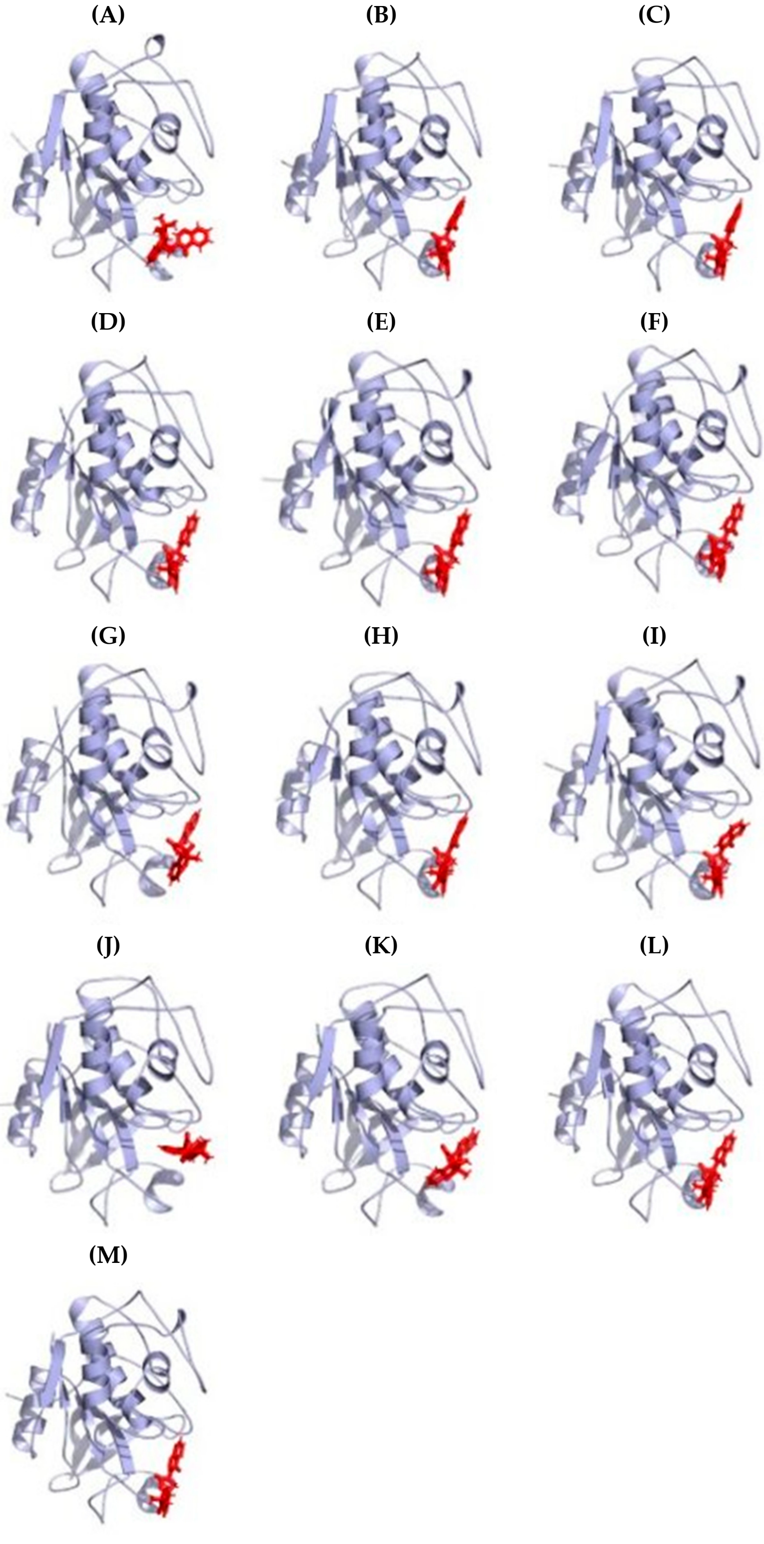

Figure S8. Representative structural snapshots of the TOP1-CatK complex obtained during the 300 ns molecular dynamics simulation. Snapshots were extracted at regular 25 ns intervals to visualize ligand-binding persistence and conformational changes within the cathepsin K active site throughout the simulation. The protein is shown as a cartoon representation, and TOP1 is shown as red sticks. **(A)** 0 ns, **(B)** 25 ns, **(C)** 50 ns, **(D)** 75 ns, **(E)** 100 ns, **(F)** 125 ns, **(G)** 150 ns, **(H)** 175 ns, **(I)** 200 ns, **(J)** 225 ns, **(K)** 250 ns, **(L**) 275 ns, and **(M)** 300 ns.

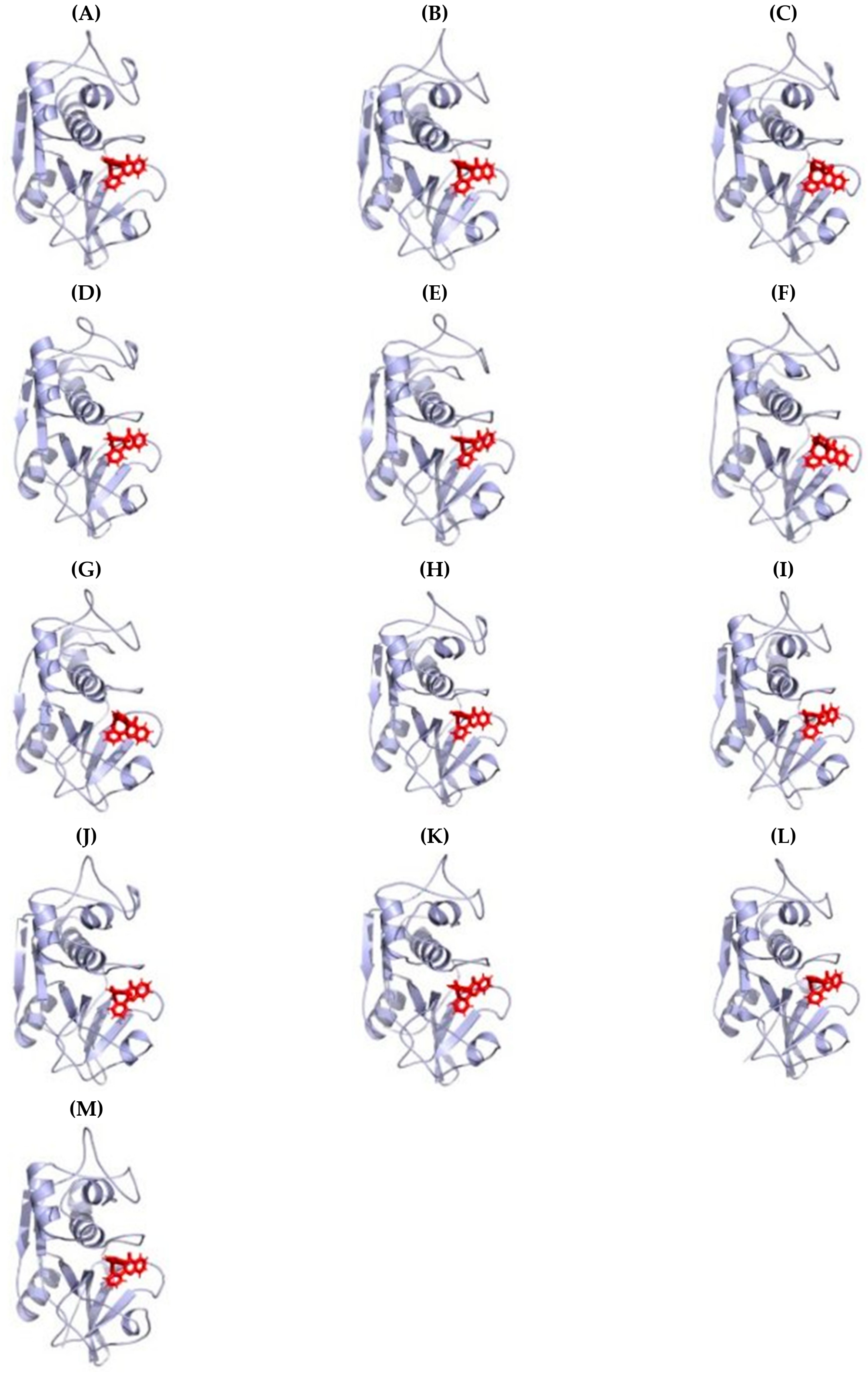

Figure S9. Representative structural snapshots of the TOP2-CatK complex obtained during the 300 ns molecular dynamics simulation. Snapshots were extracted at regular 25 ns intervals to visualize ligand-binding persistence and conformational stability within the CatK active site throughout the simulation. The protein is shown as a cartoon representation and TOP2 is shown as red sticks. **(A)** 0 ns, **(B)** 25 ns, **(C)** 50 ns, **(D)** 75 ns, **(E)** 100 ns, **(F)** 125 ns, **(G)** 150 ns, **(H)** 175 ns, **(I)** 200 ns, **(J)** 225 ns, **(K)** 250 ns, **(L)** 275 ns, and **(M)** 300 ns.

Table S1. Mean root mean square deviation (RMSD) values of cathepsin K (CatK or 2ATO) during the 300 ns molecular dynamics simulations. RMSD values were calculated for the CatK backbone atoms relative to the respective initial structures for apo CatK, the TOP1 (PubChem ID: 97043052)–CatK, TOP2 (PubChem ID: 135765825)–CatK, and reference ligand (STD, PubChem ID: 44194893)–CatK systems. Data are presented as the mean RMSD ± standard deviation (SD) in nm over the complete 0–300 ns simulation trajectory**.**

System	Mean RMSD ± SD (nm), 0–300 ns
Apo CatK	0.15 ± 0.03
TOP1–CatK	0.12 ± 0.02
TOP2–CatK	0.13 ± 0.02
STD–CatK	0.12 ± 0.01
